# Supplementary figures and images for: Beneath the surface: Amino acid variation underlying two decades of dengue virus antigenic dynamics in Bangkok, Thailand
Source: PLoS Pathog. 2022 May 2;18(5):e1010500. doi: 10.1371/journal.ppat.1010500 (PMC9098070; doi:10.1371/journal.ppat.1010500)

**DENV1**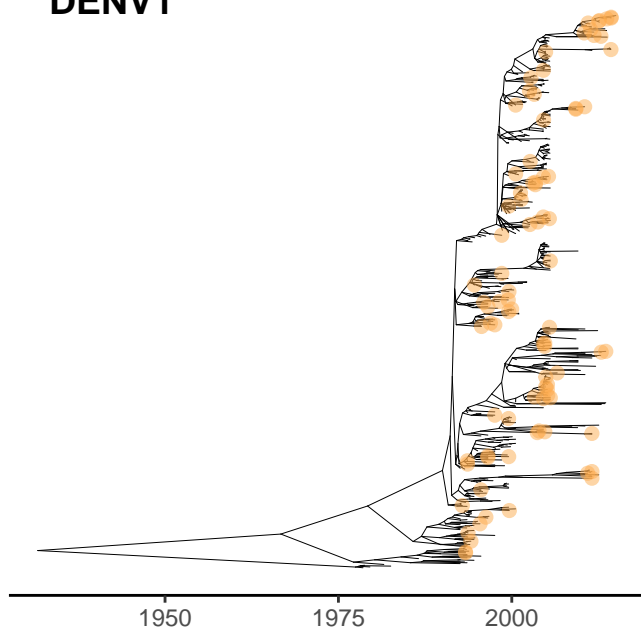**DENV2**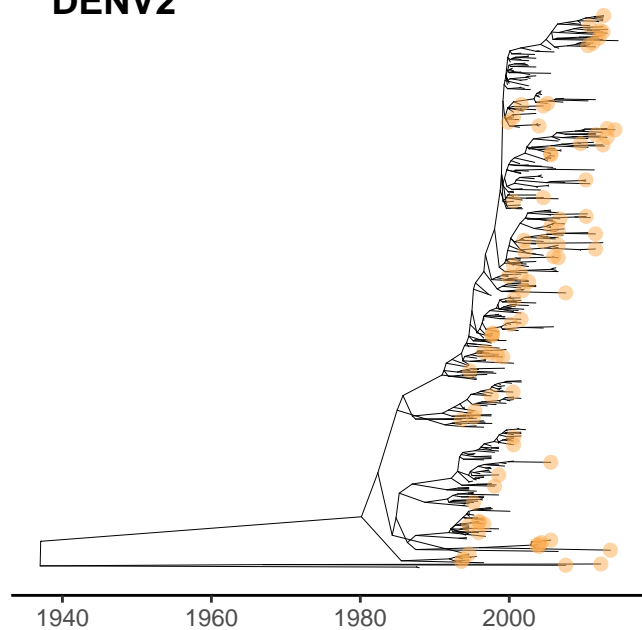**DENV3**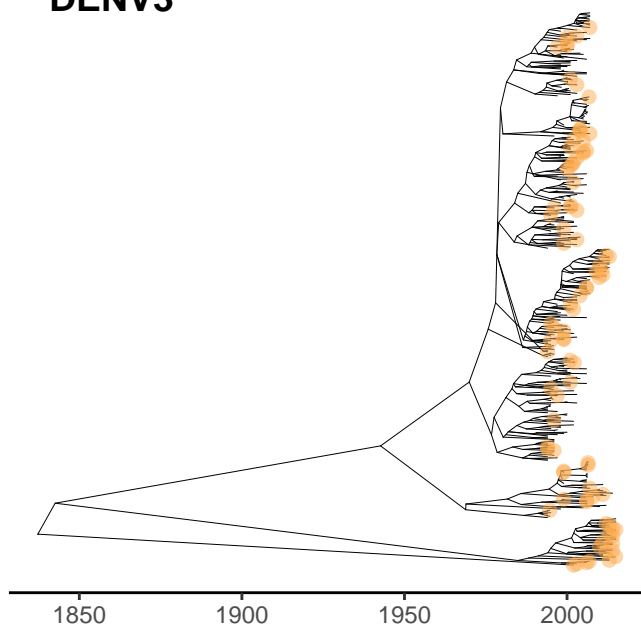**DENV4**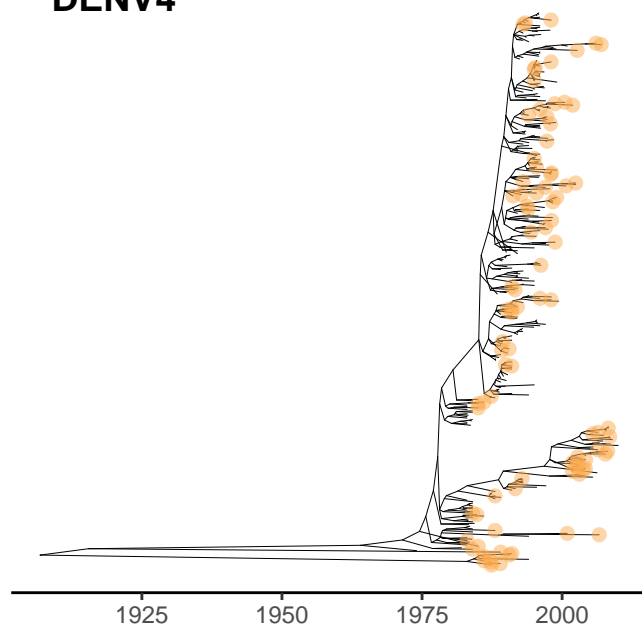

Supplement: S1 Fig — Collected from Queen Sirikit National Institute of Child Health (QSNICH) between 1994–2014. Viruses selected for antigenic characterization were marked as orange circles. (PDF) [file ppat.1010500.s001.pdf]

**a**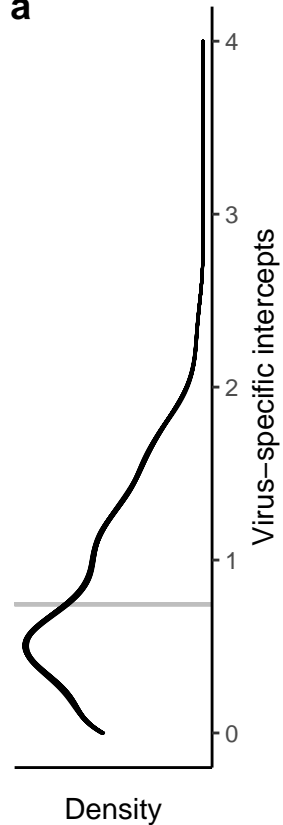**b**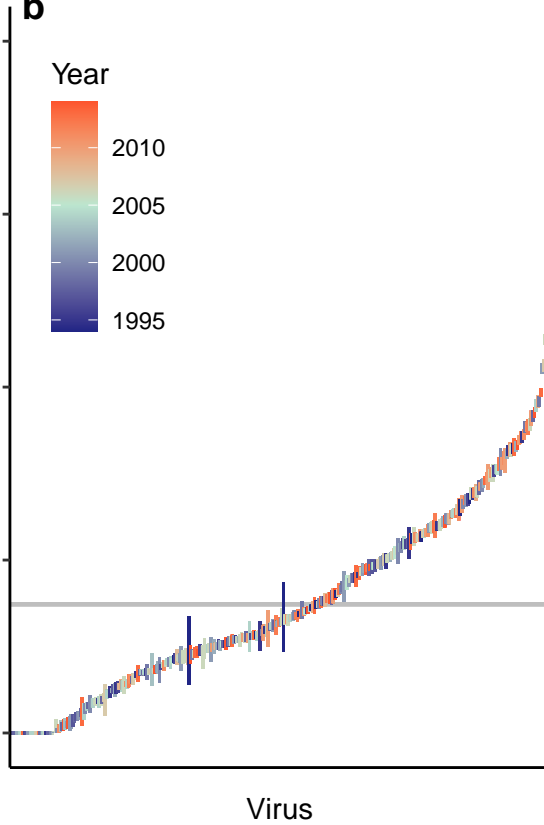**c**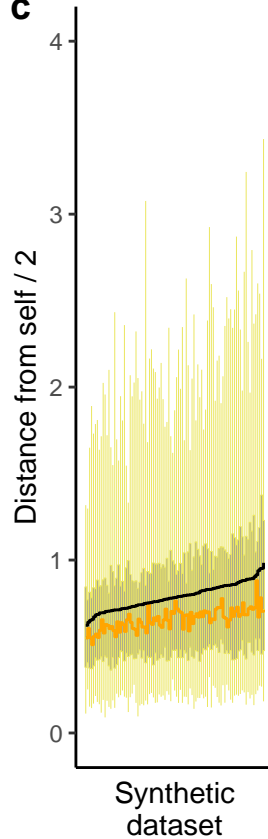

Supplement: S2 Fig — a) Distributions and b) variation in virus-specific intercepts estimated using E protein sequences across the 100 estimations. Gray horizontal lines represent the mean intercepts across viruses for each of the estimations. c) Boxplot illustrating the amount of distance attributable to measurement variability across 100 synthetic samples. Divided by two to represent the per virus contribution. Thick lines denote the means (black) and medians (orange). (PDF) [file ppat.1010500.s002.pdf]

**a**

Predicted distance

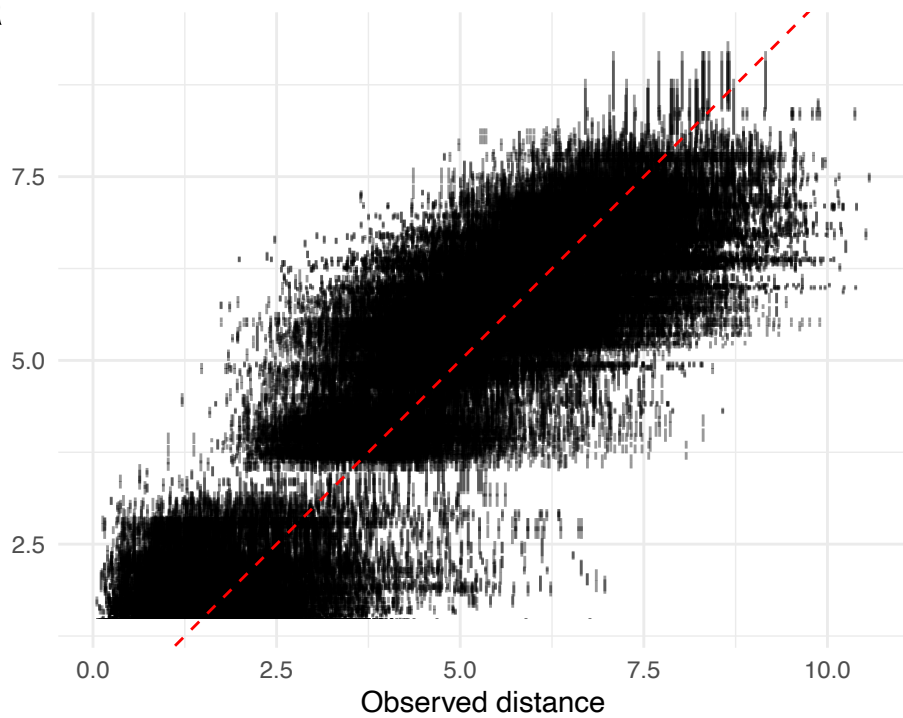**b**

Predicted distance

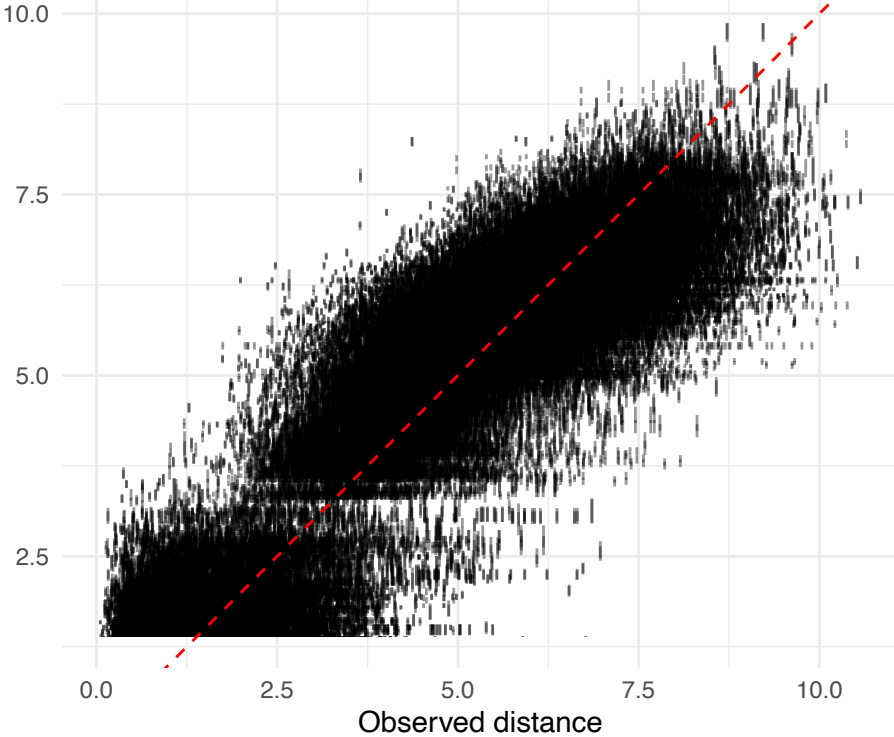

Supplement: S3 Fig — a) when effects were fitted to envelope protein sequences (E) and b) when effects were fitted to E concatenated with 62 nonzero effect sites in nonstructural protein 2A (NS2A). (PDF) [file ppat.1010500.s003.pdf]

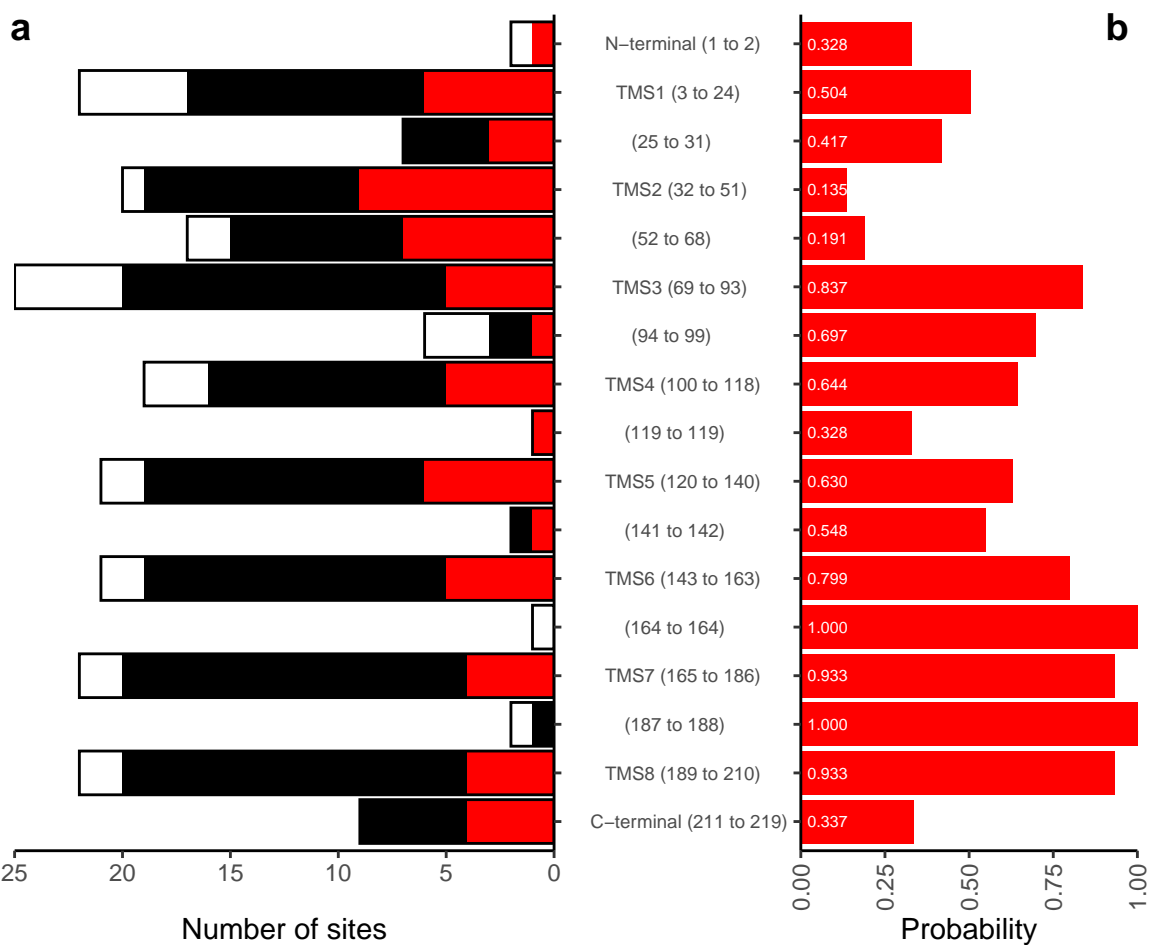

Supplement: S7 Fig — a) Total number of sites in each segment (hollow), number of variable sites (filled black), and number of sites estimated to have nonzero effects (filled red). b) Probability that at least these number of nonzero effect sites were associated with the segments at random. Amino acid positions of the segments shown in parentheses. (PDF) [file ppat.1010500.s007.pdf]

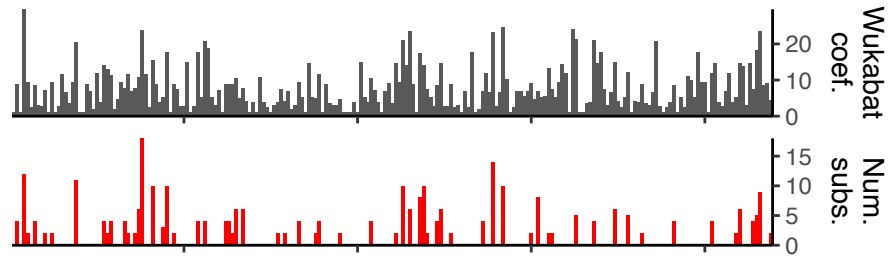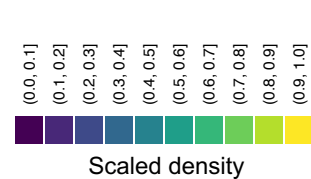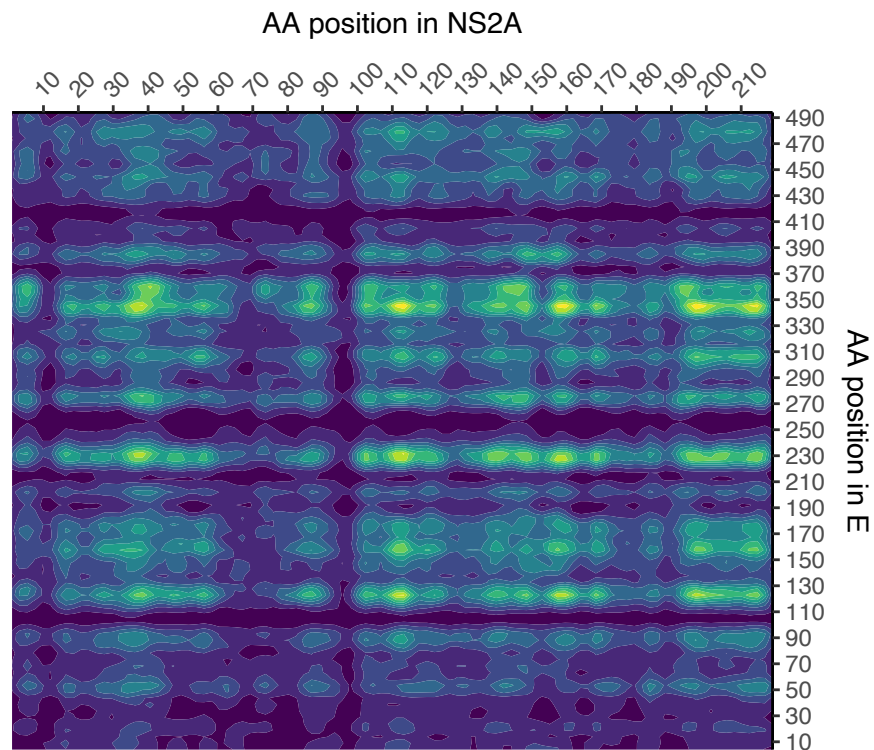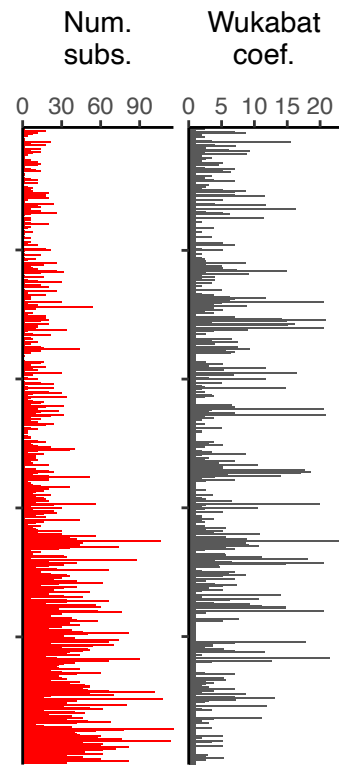

Supplement: S8 Fig — Density values were scaled to maximum value of one. Distributions of nonzero effect substitutions (red) and site-specific Wu-Kabat variability coefficient (gray) of the respective proteins are shown on top (nonstructural protein 2A, NS2A) and side (envelope protein, E). (PDF) [file ppat.1010500.s008.pdf]

**a**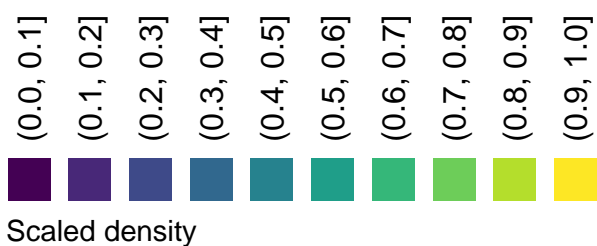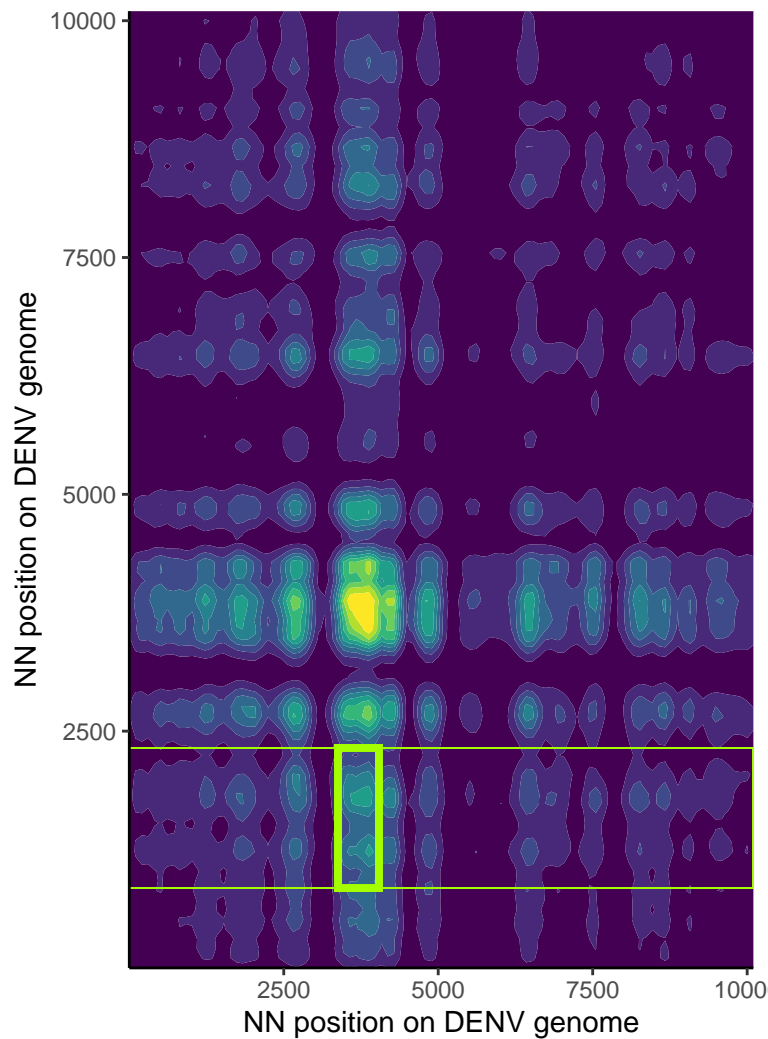**b**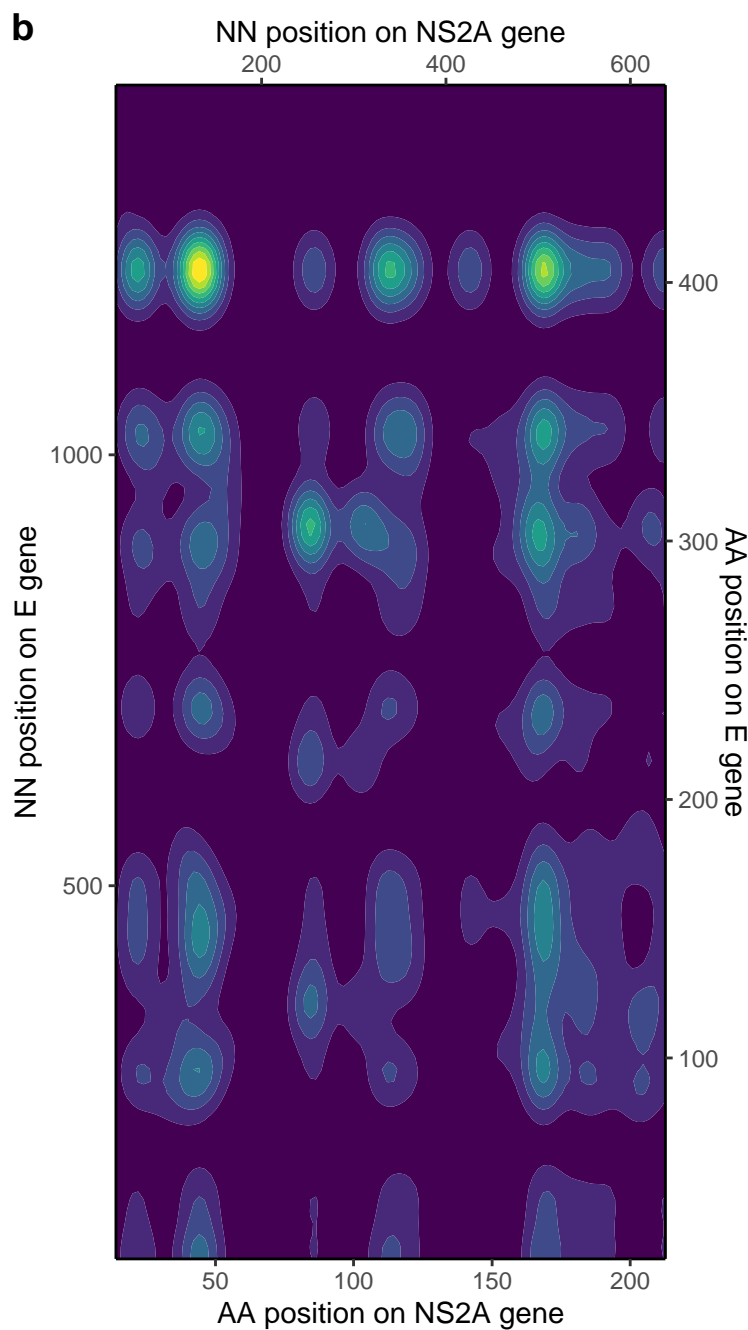

Supplement: S9 Fig — a) Density of nucleotide positions with mutual information (MI) values greater than 99th percentile of MI values between pairs throughout the DENV genome. Density scaled to maximum value of one. Thin rectangle corresponds to coevolution relationship between E gene (y-axis) and sites throughout the genome. Thick rectangle highlights relationship between E gene and NS2A gene. b) Density plot expanding the highlighted region in panel (a). (PDF) [file ppat.1010500.s009.pdf]

Substitution in E

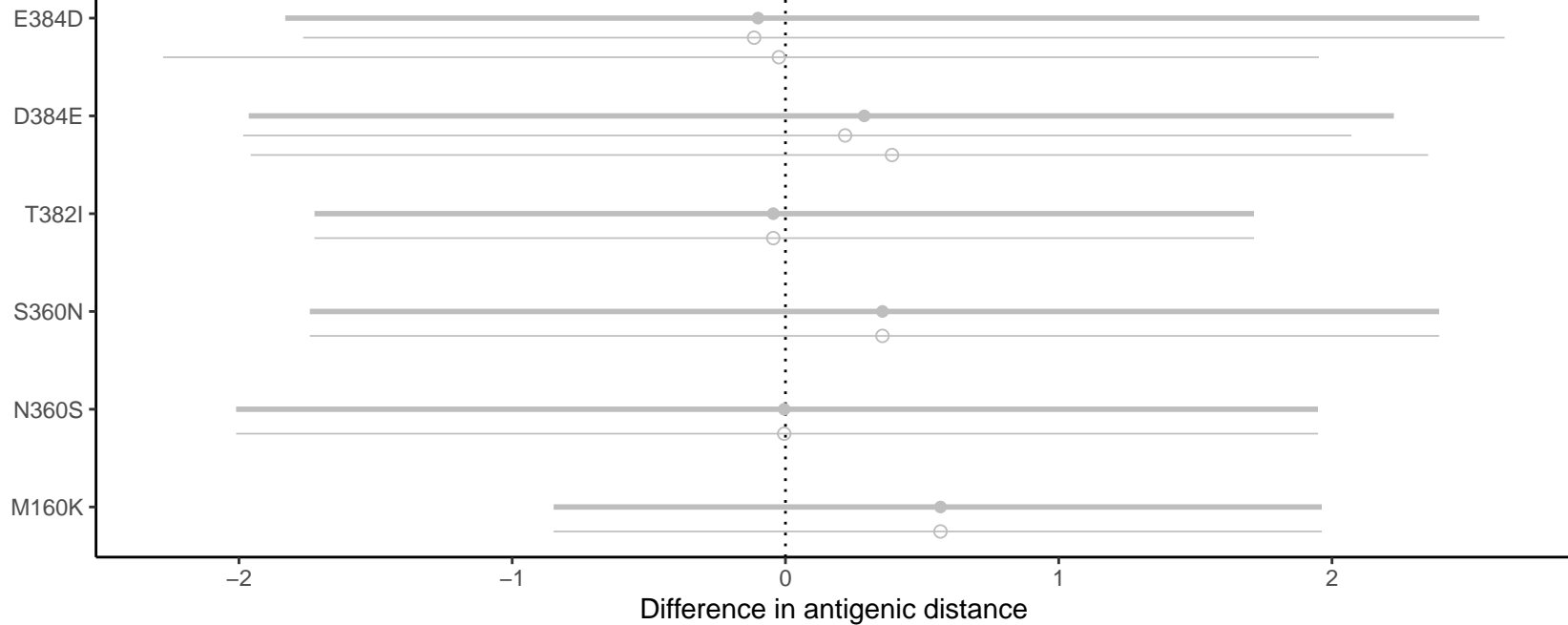

P-value

- [0,0.05]
- (0.05,0.1]
- (0.1,1]

Supplement: S11 Fig — Difference in antigenic distance observed between pairs of viruses separated by the specific substitution and antigenic distance observed in respective effectively identical viruses without the substitution (control viruses). Thick lines show median and 95% interquartile range (IQR) for triplets of all serotype pairs combined. Thin lines show the median and 95%IQR for each serotype pair identified. (PDF) [file ppat.1010500.s011.pdf]

# E: M160K

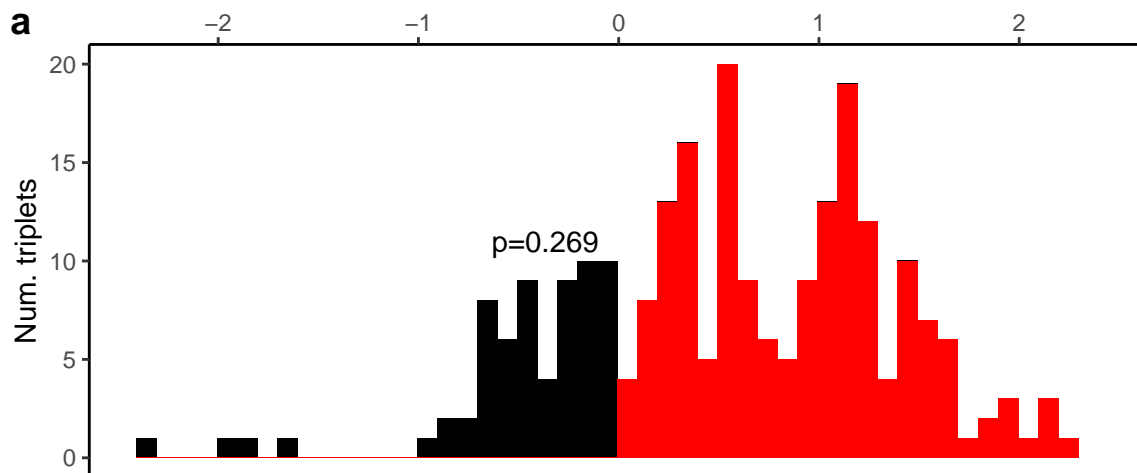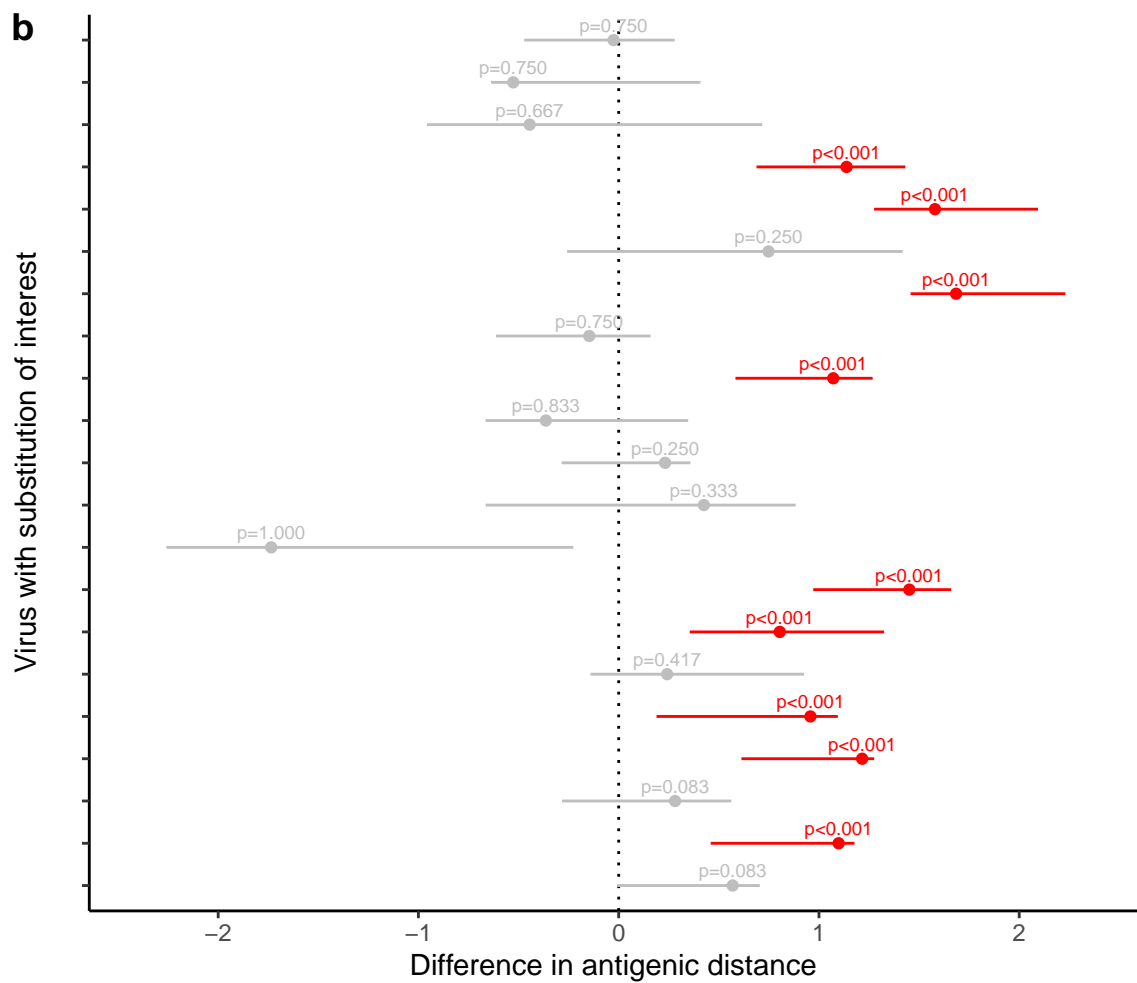

Supplement: S12 Fig — a) Distribution of difference in antigenic distance, ΔDm, for E:M160K substitution including all triplets with the same serotype pair (DENV2, DENV2) and the resultant p-value shown in comparison to b) median and 95% interquartile range of ΔDm shown separately for each virus j involved in the virus triplets and their respective p-values. (PDF) [file ppat.1010500.s012.pdf]

E: M160K  
(DENV2,DENV2)

Difference in antigenic distance

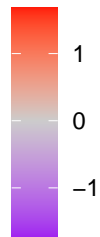

Supplement: S13 Fig — Median difference in antigenic distance, ΔDm, specific to each virus j involved in the virus triplets shown in S12 Fig are colored on the phylogeny. Points are shown as solid circles for p-values ≤ 0.05 and as hollow triangles otherwise. (PDF) [file ppat.1010500.s013.pdf]

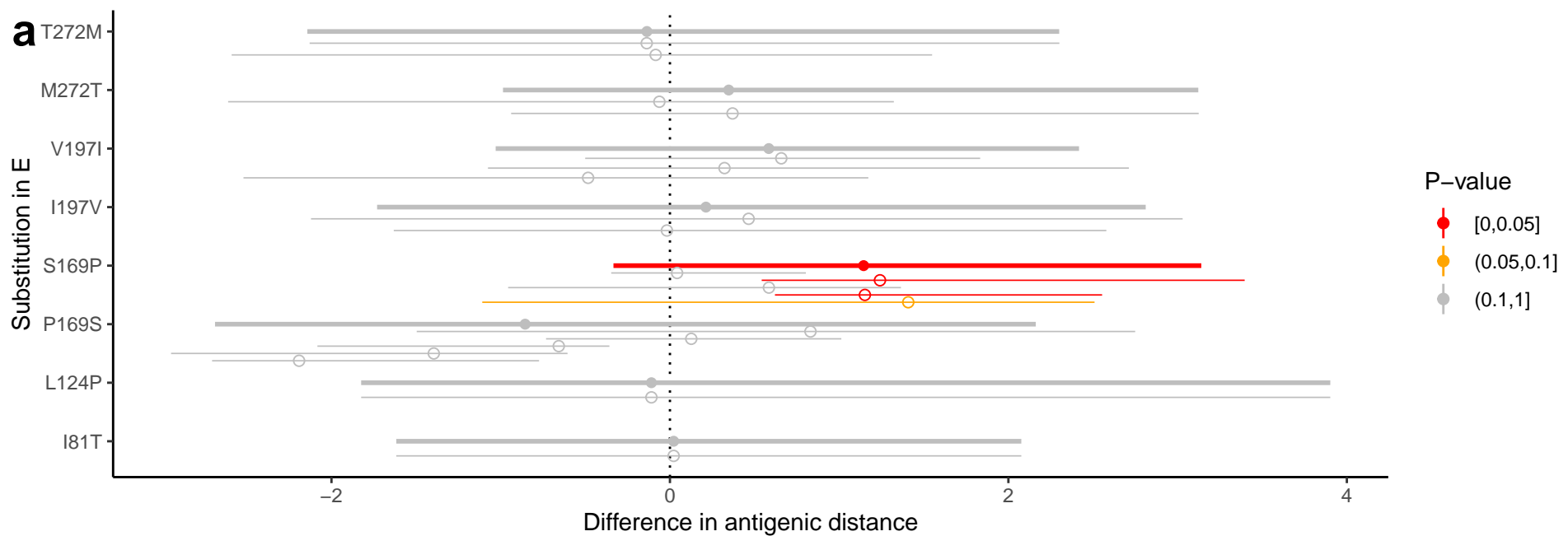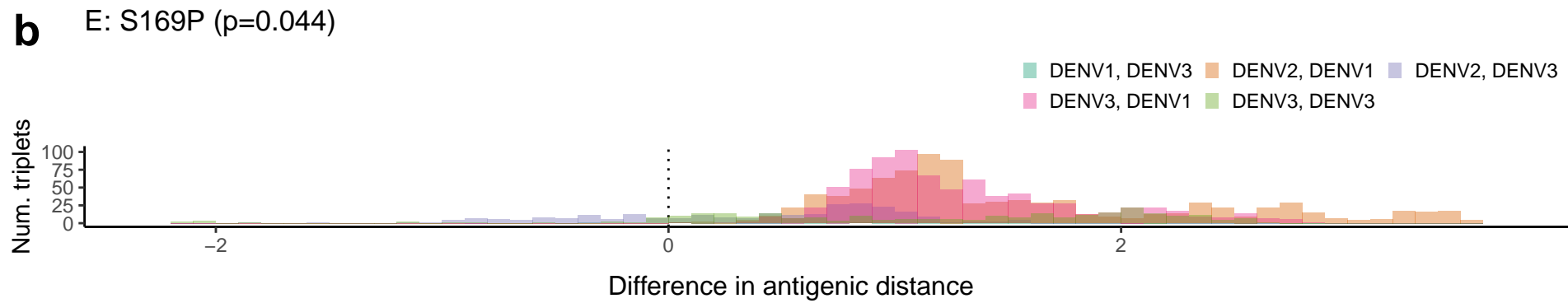

Supplement: S14 Fig — a) Difference in antigenic distance observed between pairs of viruses separated by the specific substitution and antigenic distance observed in respective effectively identical viruses without the substitution (control viruses). Thick lines show median and 95% interquartile range (IQR) for triplets of all serotype pairs combined. Thin lines show the median and 95%IQR for each serotype pair identified. b) Distribution of difference in antigenic distance for substitution with p-value ≤ 0.1 colored by serotypes of the virus pairs. (PDF) [file ppat.1010500.s014.pdf]

Substitution in E

V428M

-2

-1

0

1

2

Difference in antigenic distance

P-value

- [0,0.05]
- (0.05,0.1]
- (0.1,1]

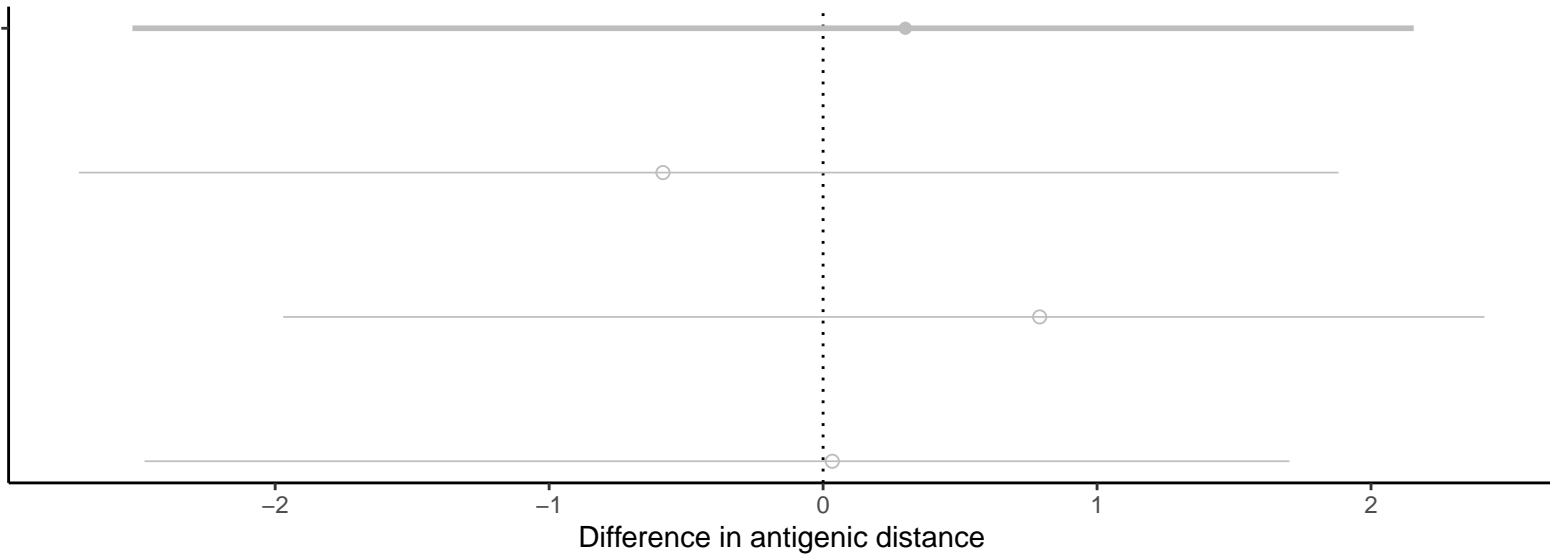

Supplement: S15 Fig — Difference in antigenic distance observed between pairs of viruses separated by the specific substitution and antigenic distance observed in respective effectively identical viruses without the substitution (control viruses). Thick lines show median and 95% interquartile range (IQR) for triplets of all serotype pairs combined. Thin lines show the median and 95%IQR for each serotype pair identified. (PDF) [file ppat.1010500.s015.pdf]

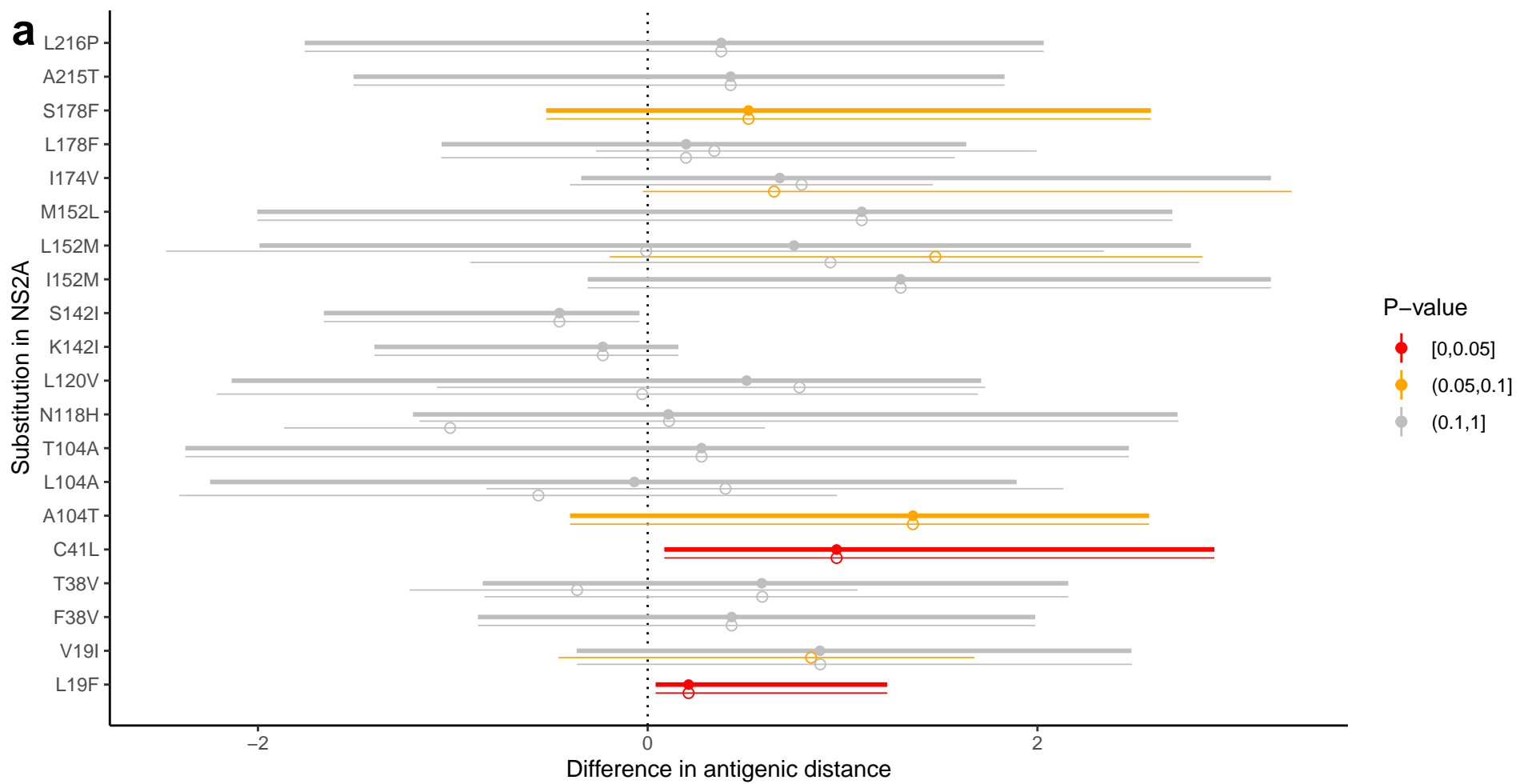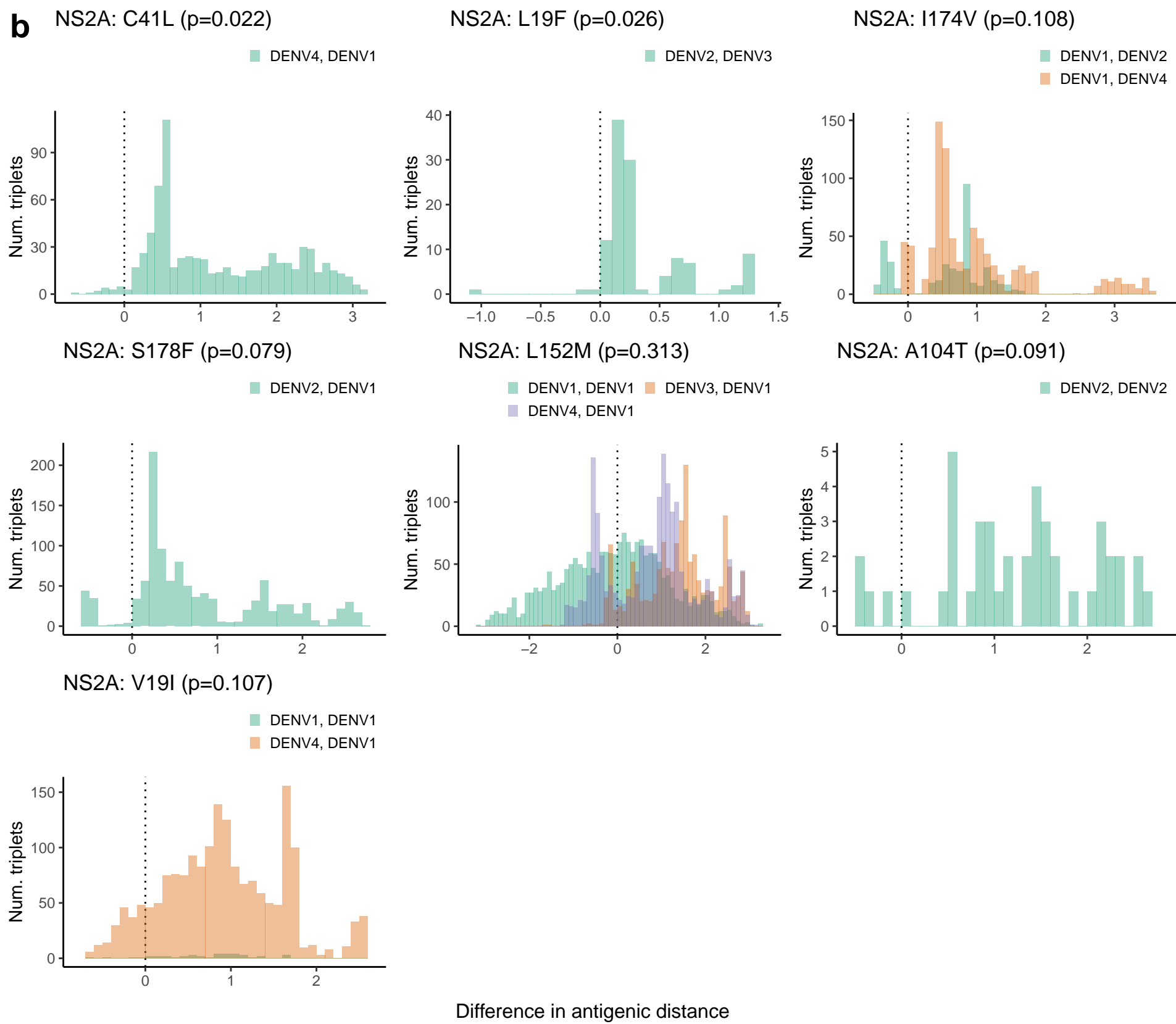

Supplement: S16 Fig — a) Difference in antigenic distance observed between pairs of viruses separated by the specific substitution and antigenic distance observed in respective effectively identical viruses without the substitution (control viruses). Thick lines show median and 95% interquartile range (IQR) for triplets of all serotype pairs combined. Thin lines show the median and 95%IQR for each serotype pair identified. b) Distribution of difference in antigenic distance for substitutions with p-value ≤ 0.1 colored by serotypes of the virus pairs. (PDF) [file ppat.1010500.s016.pdf]
