# Supplementary material for: Beneath the surface: Amino acid variation underlying two decades of dengue virus antigenic dynamics in Bangkok, Thailand
Source: PLoS Pathog. 2022 May 2;18(5):e1010500. doi: 10.1371/journal.ppat.1010500 (PMC9098070; doi:10.1371/journal.ppat.1010500)

a

	Epitope association			
	hmAb + mmAb	hmAb	Not known	Total
Nonzero effect	22 (28.6%)	21 (27.3%)	55 (71.4%)	77
Zero effect	52 (23.9%)	36 (16.5%)	166 (76.1%)	218
No diversity	37	30	163	200
Total	111	87	384	495
Odds ratio (hmAb + mmAb) : 1.28 (95%CI: 0.71, 2.29)				
Odds ratio (hmAb only) : 1.90 (95%CI: 1.02, 3.51)				

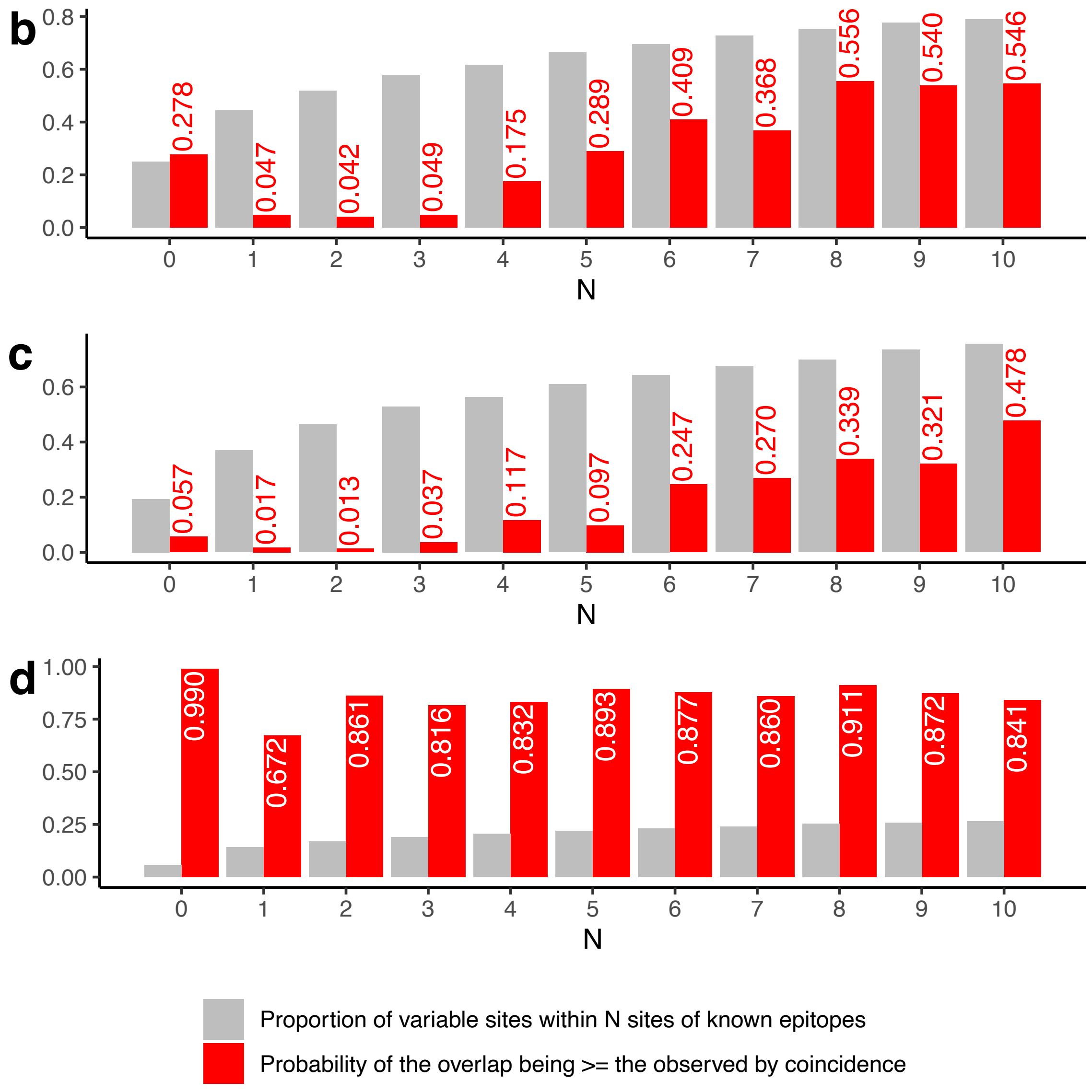

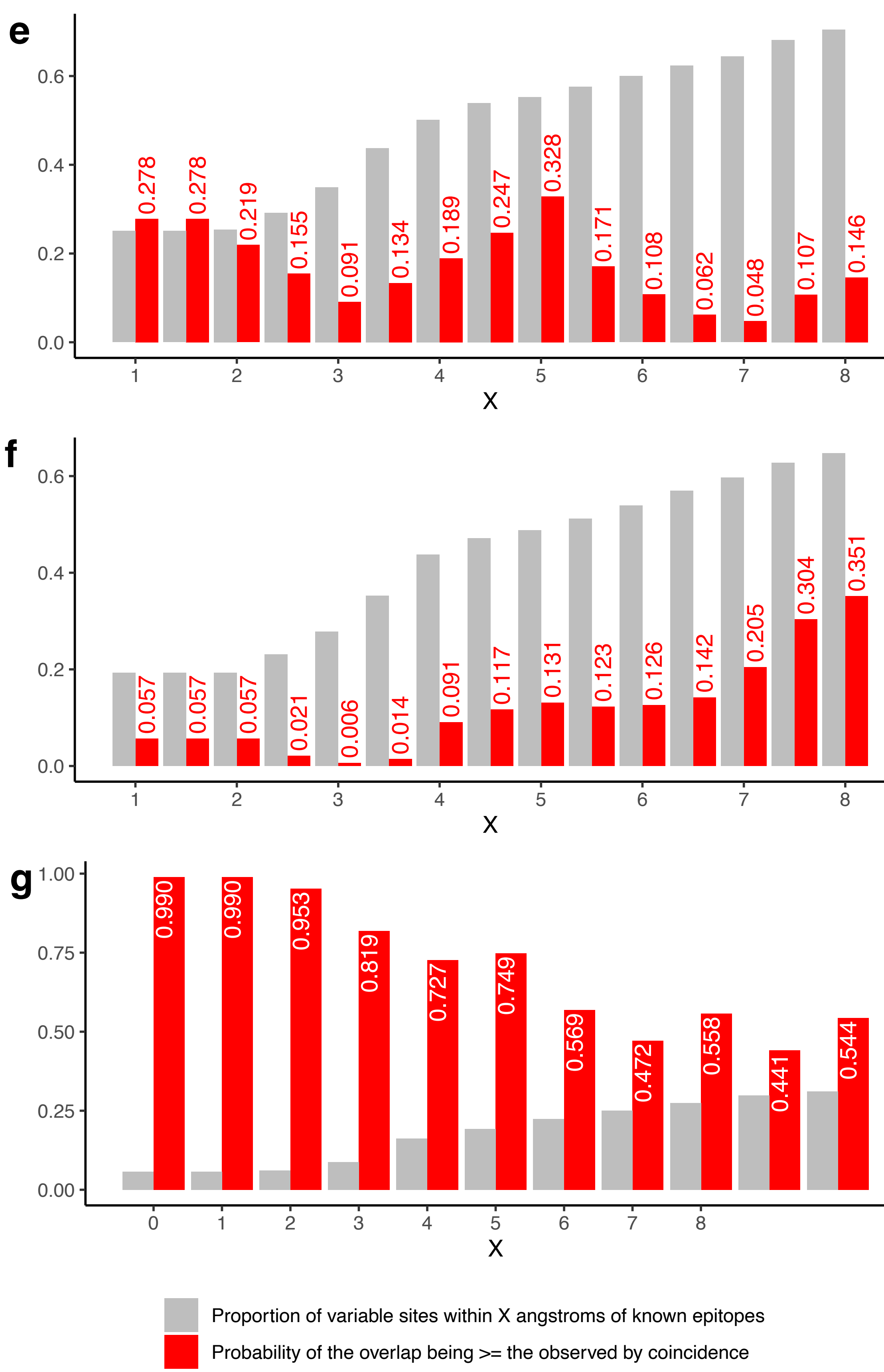

Supplement: S4 Fig — a) Number and percentage of sites with and without effects by whether or not they are part of known epitopes. Odds ratios were calculated by either considering epitopes of both human-derived monoclonal antibodies (hmAb) and murine-derived monoclonal antibodies (mmAb) and when only restricted to hmAb epitopes. Defining neighborhoods of known epitopes as positions within N sites away (linear distance), the probability of nonzero effect sites being within the neighborhood at random (red) are contrasted against the proportion of variable sites that were within the neighborhood (gray): b) known epitopes for either hmAb or mmAb, c) known epitopes for hmAb, and d) known epitopes for mmAb outside of hmAb epitopes. N = 0 was when the neighborhood was exactly at the reported epitope positions. e, f, g) Respective analogous analysis but with neighborhoods defined as being within X angstroms away from known epitopes (3-dimensional spatial distance). X = 0 was when the neighborhood was exactly at the reported epitope positions. (PDF) [file ppat.1010500.s004.pdf]
