# Supplementary material for: Beneath the surface: Amino acid variation underlying two decades of dengue virus antigenic dynamics in Bangkok, Thailand
Source: PLoS Pathog. 2022 May 2;18(5):e1010500. doi: 10.1371/journal.ppat.1010500 (PMC9098070; doi:10.1371/journal.ppat.1010500)
Supplement: S5 Fig — a) Substitutions in envelope protein (E) only, ordered by the proportion at which substitutions showed nonzero effect across the 100 estimations. Substitutions identified by our threshold of 95% was highly similar to the maximum stringency of 100%; 372/394 substitutions (94.4%). Involvement was retained in 76/77 (99%) of the sites. b) In the analysis where E was concatenated to the 62 nonstructural protein 2A (NS2A) sites which consistently showed nonzero effects in our site sampling analysis, 292/304 substitutions (96.1%) in the NS2A sites remained nonzero at a threshold of 100%. Involvement was retained in 62/62 (100%) of the sites. Proportions corresponding to nonzero effect substitutions reported in our study (threshold of 95%) are colored red. (PDF) [file ppat.1010500.s005.pdf]

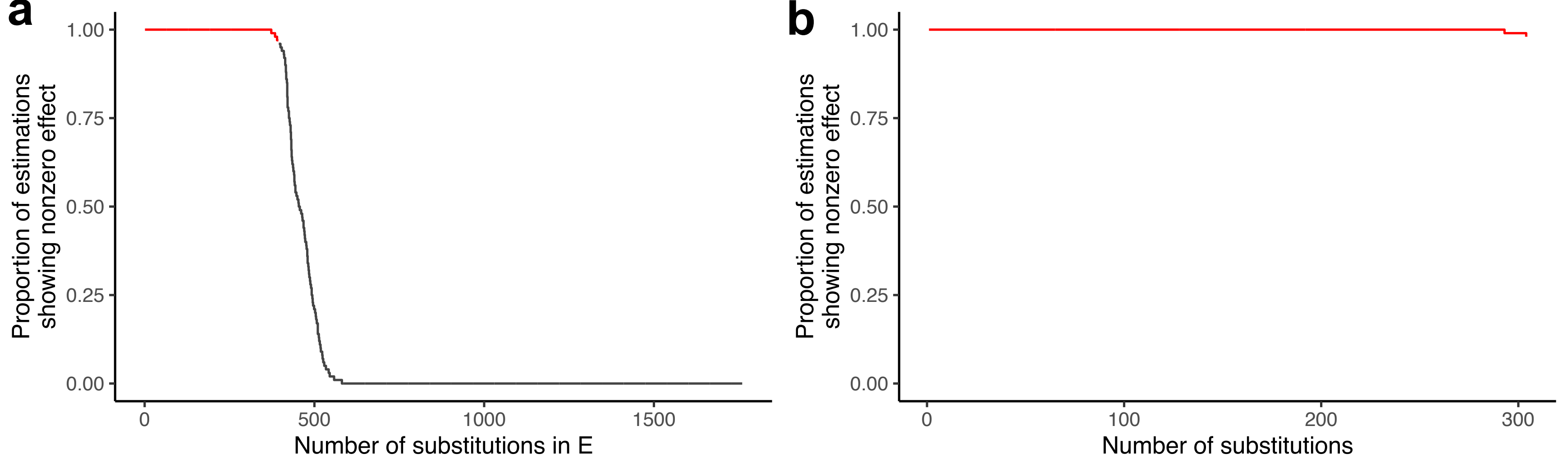
a
b
Proportion of estimations showing nonzero effect
1.00
0.75
0.50
0.25
0.00
0
500
1000
1500
Number of substitutions in E
100
200
300
Number of substitutions
