# Supplementary material for: Beneath the surface: Amino acid variation underlying two decades of dengue virus antigenic dynamics in Bangkok, Thailand
Source: PLoS Pathog. 2022 May 2;18(5):e1010500. doi: 10.1371/journal.ppat.1010500 (PMC9098070; doi:10.1371/journal.ppat.1010500)
Supplement: S6 Fig — Median effect size of substitutions across the 100-fold Monte Carlo cross-validations shown as points, 95% interquartile range as whiskers. Points are colored by locations of the sites: ER lumen (green), transmembrane (yellow), or cytosol (blue). Locations of the sites and domain annotations were taken from [34]. (PDF) [file ppat.1010500.s006.pdf]

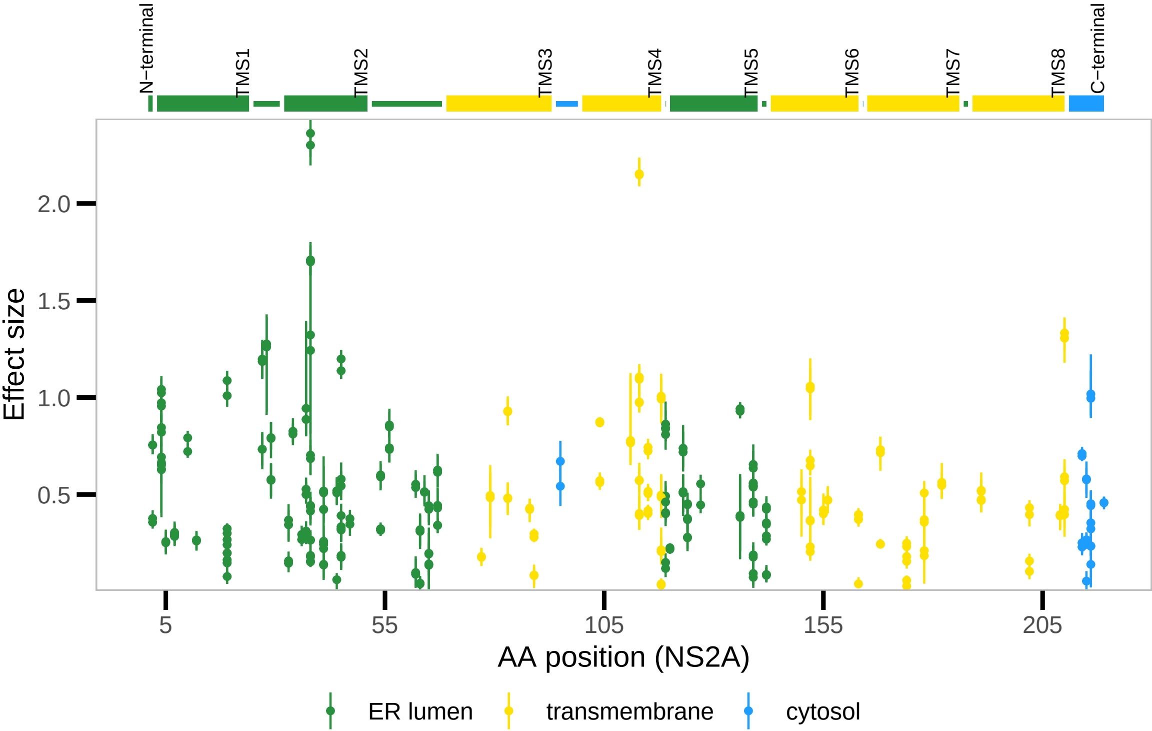
N−terminal
TMS1
TMS2
TMS3
TMS4
TMS5
TMS6
TMS7
TMS8
C−terminal
Effect size
2.0
1.5
1.0
0.5
5
55
105
155
205
AA position (NS2A)
ER lumen
transmembrane
cytosol
