# Supplementary material for: Beneath the surface: Amino acid variation underlying two decades of dengue virus antigenic dynamics in Bangkok, Thailand
Source: PLoS Pathog. 2022 May 2;18(5):e1010500. doi: 10.1371/journal.ppat.1010500 (PMC9098070; doi:10.1371/journal.ppat.1010500)
Supplement: S10 Fig — Shown separately for substitutions located in epitopes of human-derived monoclonal antibodies (hmAb), E domain I/II/III but outside of known epitopes (EDI/II/III), E stem/anchor domain, and nonstructural protein 2A (NS2A). Points are the medians of the observations/estimates. Lines are 95% interquartile ranges. (PDF) [file ppat.1010500.s010.pdf]

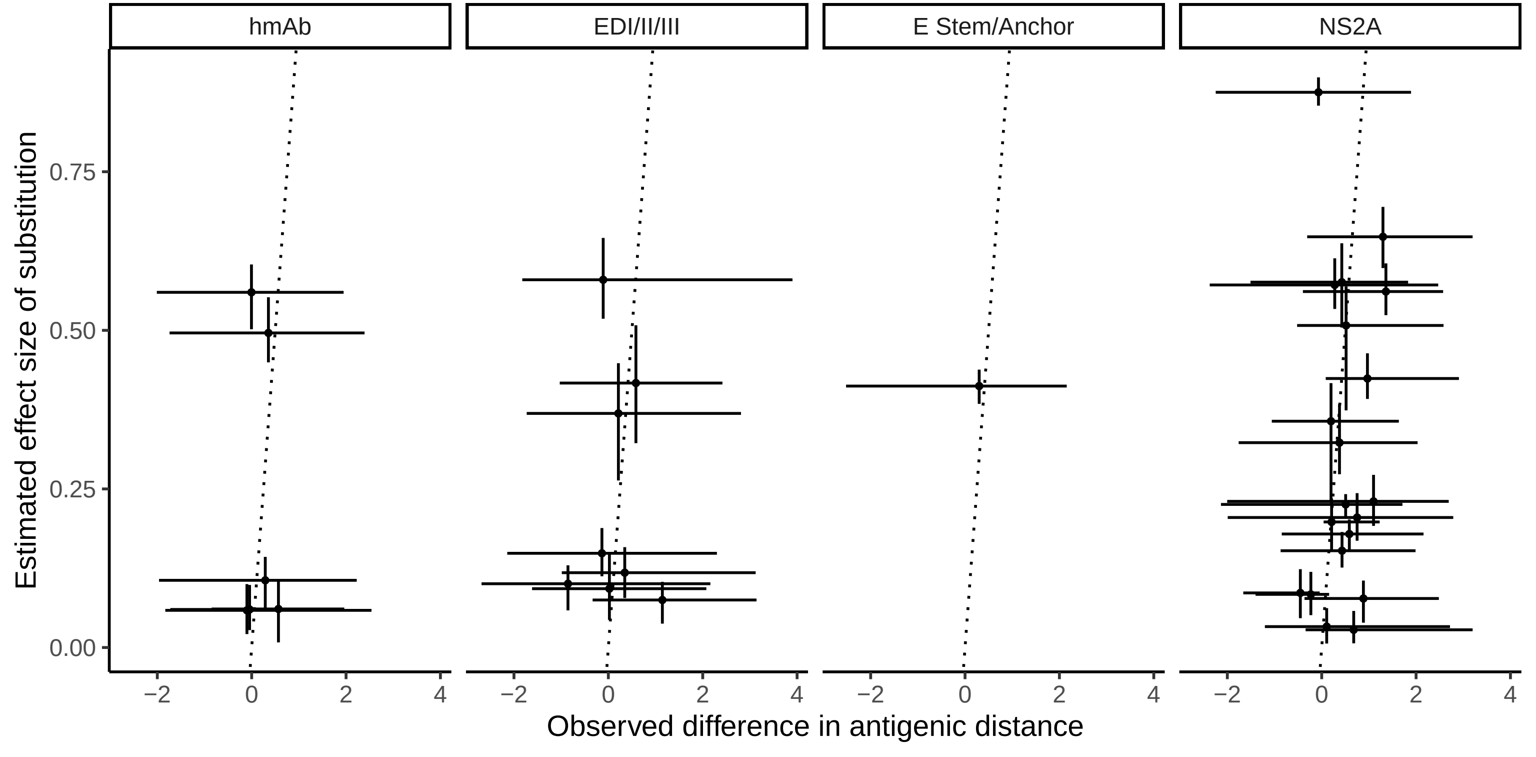
hmAb
EDI/II/III
E Stem/Anchor
NS2A
Estimated effect size of substitution
0.00
0.25
0.50
0.75
−2
0
2
4
Observed difference in antigenic distance
