# Supplementary material for: Beneath the surface: Amino acid variation underlying two decades of dengue virus antigenic dynamics in Bangkok, Thailand
Source: PLoS Pathog. 2022 May 2;18(5):e1010500. doi: 10.1371/journal.ppat.1010500 (PMC9098070; doi:10.1371/journal.ppat.1010500)
Supplement: Attachment — Submitted filename: DengueAntigenicGenetics_ResponseToReviewers.pdf [file ppat.1010500.s019.pdf]

National Institute of Allergy and Infectious Diseases
National Institutes of Health
Building 33, Room 3E19A.2, 33 North Drive
Bethesda, MD 20892-3203

March 14, 2022

PLOS Pathogens
1265 Battery Street, Suite 200
San Francisco, CA 94111
United States

Dear Drs. Lowen, Fernandez-Sesma, Haldar, and Malim,

Thank you for the opportunity to revise our manuscript, entitled "Beneath the surface: Amino acid variation underlying two decades of dengue virus antigenic dynamics in Bangkok, Thailand", for potential publication in *PLOS Pathogens*. We are grateful for your suggested edits as well as the three referees' comments. We have revised the manuscript according to the reviewers' suggestions and have included additional figures and analyses.

With respect to the request for experimental validation of the newly identified antigenic sites, we have taken advantage of the redundancy in our dataset to identify the effects of individual mutations. These analyses are detailed in numerous additional supplemental figures and in our results and discussion. However, the request to test the mutations in NS2A experimentally was not possible within the period allowed for revision of the present manuscript. Infectious clones are needed to test the effects of non-structural proteins on antigenic characteristics. Given that the strong-effect residues we observe depend on the genetic background and other substitutions, these experimental studies should be conducted using infectious clones for the strains circulating in Thailand. These experiments require designing new infectious clones, which are unusually difficult to create for flaviviruses. At present, none of the main authors on this manuscript have an infectious clone system established in their laboratories, even for prototype viruses which are mostly old, highly lab-adapted strains unrelated to the Thai viruses under examination here.

Our detailed responses to the reviewers' comments are included point-by-point below. We believe that these revisions have greatly strengthened the manuscript. Additionally, we have provided both a version of the manuscript with track changes and a clean version.

Best wishes,

Leah C. Katzelnick
Laboratory of Infectious Diseases
National Institute of Allergy and Infectious Diseases
National Institutes of Health

Derek A.T. Cummings
Department of Biology
Emerging Pathogens Institute
University of Florida

Response to reviewers

Part I - Summary

Please use this section to discuss strengths/weaknesses of study, novelty/significance, general execution and scholarship.

Reviewer #1: Huang et al. use a dataset of paired full genome dengue virus sequences with PRNT assays to measure the antigenic distance between pairs of viruses using antigenic cartography, and modify a previously published model to estimate the effects of amino acid changes that contribute to those antigenic distances. They find that sites in the E protein that are within 3 amino acids away from previously identified antibody footprints may contribute to antigenicity, as well as sites in NS2A. The method the authors use to look at combinations of sites in NS2a while controlling for association with E is clever. This paper builds nicely off of previous reports of dengue antigenic dynamics, and provides new and interesting data about the roles of substitutions beyond the E protein in antigenic variation. Overall, I think that the results are sound and that the authors' findings are novel and interesting. However, the paper would benefit from more information in the Methods, and clarification of a few points throughout the manuscript to make the paper more readily understandable for the virology audience of this journal.

Response: We thank the reviewer for these positive comments.

Reviewer #2: Huang et al. utilized a large dataset of genome sequences and antigenic information (Katzelnick et al. Science 2021), to evaluate the genetic determinants of dengue virus antigenic diversification. They found that 77 of 295 positions with residue variability in the E protein conferred antigenic effects, with only 22 of them (~28%) mapping to known epitopes, thus expanding the number of residues involved in antibody recognition/responses. This information is very interesting and could inform vaccine development. By examining the role of the other 9 dengue virus proteins, they found that the nonstructural (NS) protein NS2A presented a signal for the antigenic diversity detected at antibody level with neutralization assays. They performed different analyses and tested different hypotheses to show that the role of antigenic diversification of NS2A is not linked to similar ancestries on the genome. The groups collaborating on this study are leaders on dengue epidemiology and immunity and the data is of interest, but there is no real explanation on how this NS protein is involved on antibody recognition and virus neutralization. It has been shown that NS2A plays a role in virus RNA replication and potentially in the evasion of the interferon response, but there is no study showing that antibodies are directed to the NS2A from dengue virus, making it difficult to develop a coherent explanation for these results.

Response: We thank the reviewer for these thoughtful comments. We have added additional analyses to our manuscript, detailed in response to the reviewer's comments below.

Reviewer #3: Huang et al., examine the genetic variation among dengue viruses (DENV) from historic samples in Thailand that span several years. The authors examine the relationships between amino acid residue variation within and outside the Envelope protein, a target of neutralizing antibodies, in potentially modulating the neutralizing activity of antibodies. Overall, the authors report several interesting, hypothesis-generating observations. However, their findings fall short as they do not validate any of their descriptive analyses.

Response: We thank the reviewer for this feedback. The manuscript has been revised to include additional evaluation of our initial observations, explained in detail below.

Part II - Major Issues: Key Experiments Required for Acceptance

Please use this section to detail the key new experiments or modifications of existing experiments that should be absolutely required to validate study conclusions.

Generally, there should be no more than 3 such required experiments or major modifications for a "Major Revision" recommendation. If more than 3 experiments are necessary to validate the study conclusions, then you are encouraged to recommend "Reject".

Reviewer #1: (No Response)

Reviewer #2: 1. The data involving NS2A on dengue virus antigenic diversification is interesting but speculative. The authors are from multiple established and well-funded laboratories and should be able to provide experimental data rather than "inviting assessment of these effects in vitro" or state that "it would be interesting to see how the effects compare when introducing these substitutions into other genetic backgrounds experimentally."

Response: We thank the reviewer for the suggestion to validate our findings on NS2A. We have added an extensive new analysis to test observable effects of individual substitutions including in NS2A described as follows:

Results:

"Drawing from the existing diversity of the 348 closely-related virus strains in our dataset, we examined whether the marginal effects identified in the substitution model could be observed for viruses separated by individual substitutions. We consider viruses with identical sequences in E and the 62 nonzero effect sites in NS2A as effectively identical. With the high genetic similarity between viruses in our dataset, we were able to identify pairs of viruses that were separated by a substitution of interest (virus i vs. virus j) and a control virus that was otherwise effectively identical (virus j^c). We identified a sufficient number of these 'triplets' (i,j,j^c) to test isolated effects of six substitutions in footprints of human-derived mAb (hmAb), one substitution in EDI/II/III but outside of known mAb epitopes, eight substitutions in stem/anchor domain of E, and twenty substitutions in NS2A."

Below describes our findings pertinent to NS2A:

We also performed the triplet analyses on other sites in E and in NS2A. We found significant effects ($p \le 0.05$) for 1 of 8 substitutions in EDI/II/III but outside of known mAb epitopes (S169P, \nameref{S:triplet_e.nonstem}), 0 of 1 substitutions in stem/anchor of E (\nameref{S:triplet_e.stem}) and 2 of 20 substitutions in NS2A (L19F and C41L, \nameref{S:triplet_ns2a}). The NS2A substitution C41L is in one of the coevolution hotspots with E, and is within pTMS-2, the region most associated with antigenic effect in our larger model.

We further discussed these findings in the discussion as follows:

"To further evaluate how the observed effects hold across genetic backgrounds, we tested whether viruses with and without identified antigenic determinants in E and NS2A differ in antigenicity in the absence of other sources of antigenically relevant changes, thus drawing on the redundancy in our existing dataset to identify the antigenic effects attributable to single amino acid changes. These analyses are a prerequisite for experimental studies to test individual mutations in a clonal background. Our analysis shows that the background virus is important, suggesting experimental studies to identify substitutions driving antigenic changes should be conducted using infectious clones specific to the virus population under study. As designing infectious clones for flaviviruses is difficult, these substitutions provide best candidates for extensive studies to uncover molecular mechanisms underlying the relationship between these substitutions and changes in antigenicity of DENV."

This new analysis approach is described in detail in the Methods, "*Assessing observable isolated effects of substitutions" and in a new supplemental figure (S16 Fig):*

"To evaluate further how the specific substitutions estimated to have nonzero effects by the substitution model hold across genetic backgrounds, a suitable first step is to test whether viruses with and without these substitutions differ in antigenicity in the absence of other sources of antigenically relevant changes. Thus, we queried our dataset for virus triplets to as closely simulate experimental validation using infectious clones, where each mutation would be introduced separately into clonal backgrounds. Because our

outcome measure is antigenic distance, the equivalent experiment would be to take a reference virus i and measure the fold-difference in titers across all sera in the serum panel to virus j. We would then do the same with control virus j^c, which is equivalent to virus j. All measures of distance are antigenic distances between pairs of viruses, which is related to the fold-drop in neutralization titers.

In our dataset of Thai DENV, we identified virus pairs (i,j) that were separated a set of substitutions M where the substitution of interest $m \in M$, then queried for control viruses j^c where substitutions separating (i, j^c) equals M-{m}. As a result of the common substitution requirement, j and j^c were always of the same serotype. For each virus triplet (i,j,j^c) identified, we compute the difference in observed antigenic distance between (i,j) and (i, j^c). We denote this difference as ΔD_m. In considering only substitutions in E and the 62 sites in NS2A, our analysis assumes that substitutions outside of E and the 62 NS2A sites do not contribute to antigenic changes. We derived the p-value in rejecting the null hypothesis that ΔD_m <=0 by calculating the proportion triplets with ΔD_m=0. As effects may be background dependent, the calculations were done separately for each serotype pair of (i,j) identified. Calculations were limited to sets of virus triplets that involved greater than two distinct viruses i and had greater than 30 triplets identified."

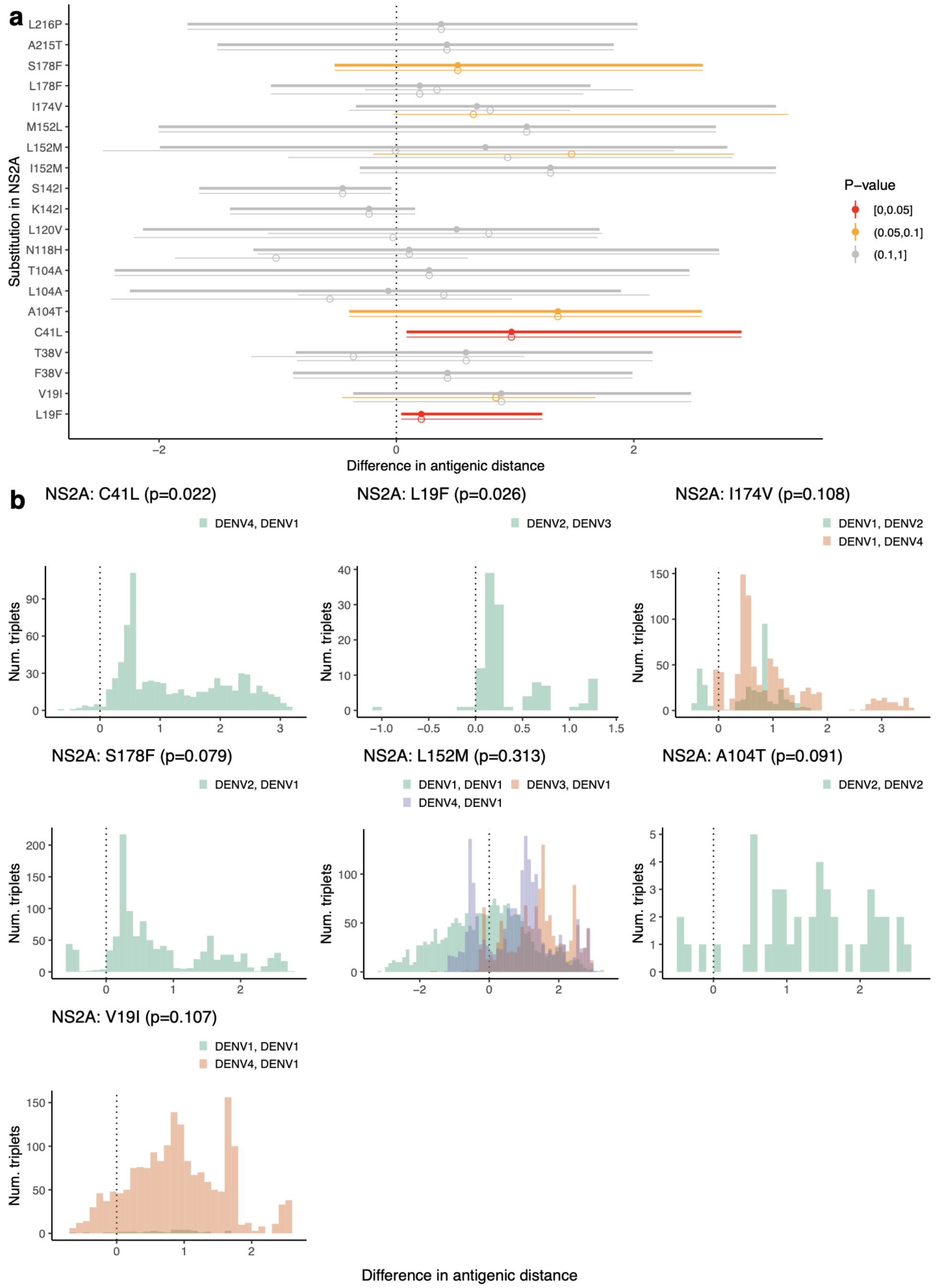

S16 Fig. Effects of substitutions in nonstructural protein 2A (NS2A). a) Difference in antigenic distance observed between pairs of viruses separated by the specific substitution and antigenic distance observed in respective effectively identical viruses without the substitution (control viruses). Thick lines show median and 95% interquartile range (IQR) for triplets of all serotype pairs combined. Thin lines show the median and 95%IQR for each serotype pair identified. **b)** Distribution of difference in antigenic distance for substitutions with p-value ≤ 0.1 colored by serotypes of the virus pairs.

2. From those 55 residues that do not map on known epitopes but predicted by the model to be involved in antigenic diversity (Fig 2), authors found that “30 were within 3 sites from known epitopes” suggesting a potential role of antibody recognition. This reviewer could not find how the authors calculated the distance from residues mapping to known epitopes. Was this calculated using linear sequences or structural information from the E protein? Please provide a better description on the methods section on how this analysis was carried out. Linear sequence information might not be the best predictor of the role of these residues on antibody recognition as “distant” residues could be “close” when the structural information is considered. Again, authors have all the resources to provide experimental data to confirm whether those 55 residues that do not map on known epitopes are involved in antigenic diversity.

Response: Thank you for this comment. We added language to clarify that the distances were based on linear sequences. In addition, we now also performed the analysis on distances from resolved 3-dimensional structures and have added these results to the main text. The revised text to the following.

> *“Interestingly, while 36 sites previously identified as DENV-specific hmAb epitopes were marked as zero-effect size by the model, 29 sites (80.5%) were within 3 linear positions away from a nonzero effect residue. In reverse, of the 56 nonzero effect sites that did not match the reported hmAb epitopes, 28 were within 3 sites of known hmAb epitopes, suggesting that they plausibly could contribute to epitopes for some previously identified antibodies. The chance of observing at least this amount of overlap, 21 captured + 28 proximal, if 77 sites were chosen from the 295 sites with variability at random was small (p=0.037, Fig 2b). We repeated the analysis using distances extracted from a resolved 3-dimensional structure of E [28]. The chance was also small when proximal sites were defined as being within 3.5 angstroms away (p=0.014, Fig 2c).”*

We further added the calculation methods as follows.

> ***Assessing association between effect sites and known epitopes***
> *For a set of epitope neighborhood sites M and a set of nonzero effect sites S, the observed number of overlap between them equals $|M \cap S|$. If $|S|$ nonzero effect sites were sampled from the set of variable sites V at random, we would expect the proportion of overlap p to be $|M \cap V|/|V|$. Because effects can only be attributed to variable sites, $S \subset V$, it follows that $|S \cap V| = |S|$ and $|M \cap S \cap V| = |M \cap S|$. The binomial probability of observing an overlap of at least $|M \cap S|$ if S was sampled from V at random equals*

$$\sum_{u=|M \cap S|}^{|S|} \binom{|S|}{u} p^u (1-p)^{|S|-u}$$

3. The association of the NS2A protein to antibody recognition and virus antigenic diversification could be linked to interactions between these two proteins, E and NS2A, at the replication level.

Authors could use phylogenetic methods and additional sequences from other studies to determine whether this signal is associated to co-evolution.

Response: Thank you for this suggestion. We have now added the proposed analyses based on our review of available methods for coevolution analysis between sites. We included these results in the coevolution of E-NS2A sites section in the main text as follows:

> ***E-NS2A coevolution hotspots supports interprotein interactions***
> *Coevolution between sites may indicate interprotein interactions [40]. To explore whether antigenic signals in NS2A could be linked to interactions with sites in E, we applied two coevolution detection methods to our dataset. The first method, fastcov [41], retains both site and residue information and takes into account asynchronous changes at different sites. The use of covariance between sites in the method has been shown to correspond well with branching patterns in the phylogeny. S8 Fig illustrates the density of coevolving residue pairs between sites in E and NS2A identified by fastcov. The second method, SpydrPick [42], is a mutual information (MI) based method with phylogenetic signal adjustment that detects coevolution between nucleotide positions. Filtering for pairs of nucleotide positions with MI values greater than the 99th percentile across all position pairs on the genome, NS2A appears to show a comparatively high level of coevolution with E compared to other proteins (S9 Fig). The coevolution hotspots suggested by both methods were around positions 40 (pTMS-2), 115 (pTMS-4), and 160 (pTMS-6) in the NS2A protein, which coincide with regions of high diversity and the identified nonzero effect sites. These results suggest possible interactions between E and NS2A at these sites, making substitutions in these hotspots interesting candidates for follow up in-depth investigation.*

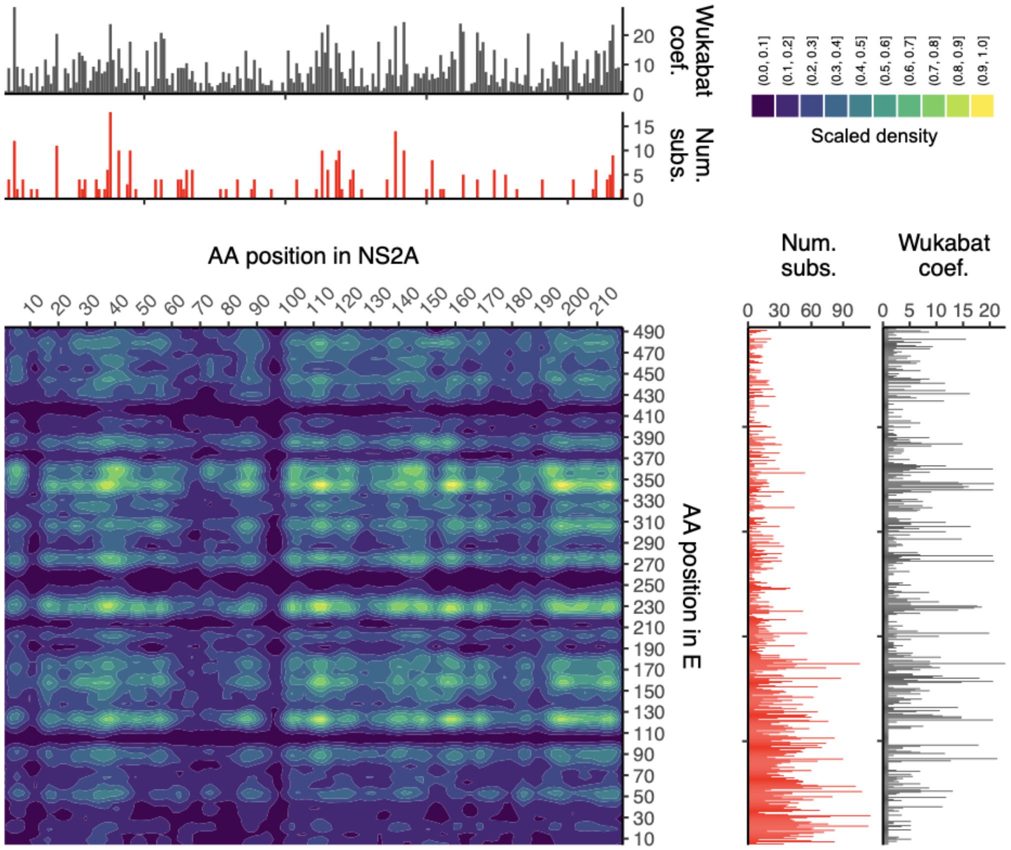

S8 Fig. Density of coevolving residue pairs detected by *fastcov*. Density values were scaled to maximum value of one. Distributions of nonzero effect substitutions (red) and site-specific Wu-Kabat variability coefficient (gray) of the respective proteins are shown on top (nonstructural protein 2A, NS2A) and side (envelope protein, E).

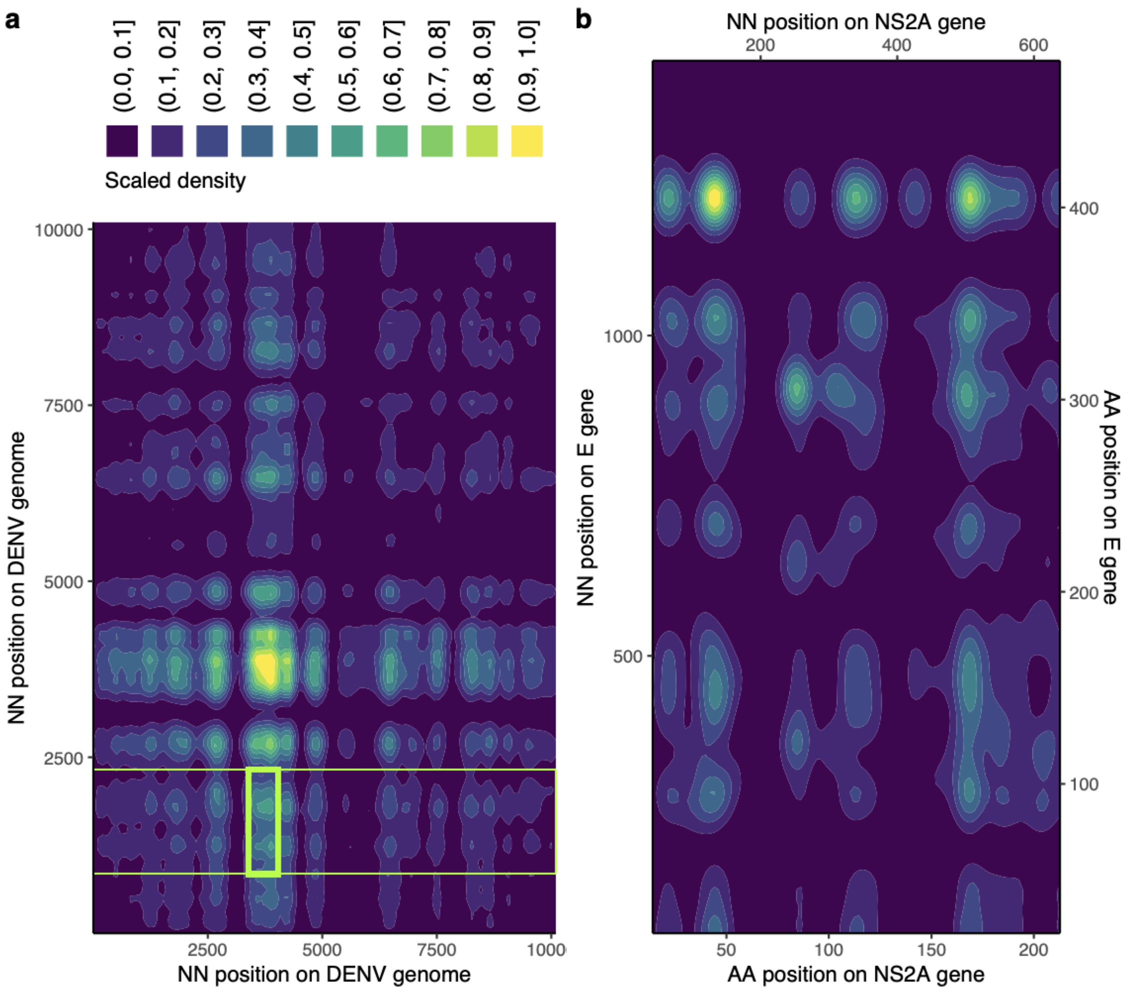

S9 Fig. Density of coevolving nucleotide pairs detected by *SpydrPick*. a) Density of nucleotide positions with mutual information (MI) values greater than 99th percentile of MI values between pairs throughout the DENV genome. Density scaled to maximum value of one. Thin rectangle corresponds to coevolution relationship between E genes (y-axis) and sites throughout the genome. Thick rectangle highlights relationship between E gene and NS2A gene. **b)** Density plot expanding the highlighted region in panel (a).

Reviewer #3: 1. Have the authors validated any of the Envelope AA residues that are outside of the mAb footprints with respect to having an impact on neutralizing antibodies? Are there viral isolates available or are there recombinant viruses that can be used to validate some of their findings in neutralization assays with specific monoclonal antibodies? There are several hits from EDI and EDII that came up on their nonzero effect size. While the computational data is interesting and potentially compelling, it would be good to validate the data with these well characterized mAbs: 1F4, 14C10, 2D22, and 5J7, EDE1-2B2, and EDE1-2C8.

Response: Thank you for this suggestion. We have developed a new analysis to evaluate the individual effects of specific mutations using diversity present in our dataset: It is described in the methods section (Assessing observable isolated effects of substitutions) as well as in the Results and Discussion:

Results:

> *"Drawing from the existing diversity of the 348 closely-related virus strains in our dataset, we examined whether the marginal effects identified in the substitution model could be observed for viruses separated by individual substitutions. We consider viruses with identical sequences in E and the 62 nonzero effect sites in NS2A as effectively identical. With the high genetic similarity between viruses in our dataset, we were able to identify pairs of viruses that were separated by a substitution of interest (virus i vs. virus j) and a control virus that was otherwise effectively identical (virus j^c). We identified a sufficient number of these 'triplets' (i, j, j^c) to test isolated effects of six substitutions in footprints of human-derived mAb (hmAb), one substitution in EDI/II/III but outside of known mAb epitopes, eight substitutions in stem/anchor domain of E, and twenty substitutions in NS2A. No nonzero effect substitutions in footprints of murine-derived mAb (mmAb) but outside of hmAb footprints had sufficient virus triplets for evaluation. The number of virus triplets were primarily limited by low number of control viruses due to coupling of the substitutions with other substitutions (470/698 substitutions). Notably, of the strongest effect sizes observed in our models, 138/229 substitutions with effect size >0.5 were not testable with the triplet analysis because these mutations were often accompanied by other antigenically important changes.*
>
> *We found broad correspondence between differences in antigenic distances observed from virus triplets and effect sizes estimated by our model in all substitution groups (S10 Fig). For all substitutions, differences in antigenic distance observed from virus triplets (ΔD_m) have wide 95%IQR. Given that we have matched for all changes in E and the 62 NS2A sites, we suspect that the wide confidence intervals are due to smaller sample sizes of 'testable' triplets. This validation is thus likely underpowered and cannot overcome variability of the measurements, an issue that would also likely affect experimental studies introducing individual mutations synthetically into infectious clones. We found that none of the 6 testable substitutions in footprints of hmAb had significant effects (S11 Fig). However, the genetic background had an important effect on the significance of each triplet. Take for example a substitution in the footprint of hmAbs on E, M160K, which has been shown experimentally to have a modest antigenic*

effect [43]). S12 Fig contrasts the overall distribution of ΔD_m for M160K against ΔD_m associated with each virus tested individually. Nearly half of the individual viruses have significant effects, and these effects are clustered when mapped to the phylogeny, indicating the effect is dependent on the background genome (S13 Fig). This suggests that the particular virus this mutation is introduced into will affect the magnitude of the antigenic effect observed, even when working with closely related viruses of the same genotype circulating in a single city over time.

In the few substitutions that involved multiple serotype pairs, effects of the substitutions appeared to vary by serotype. For instance, albeit significant overall effects were observed for E:S169P, DENV2-DENV3 pairs were far from rejecting the null (S14Fig). This heterogeneity further suggests that the effects of substitutions are background-dependent, which also partly explains the wide variation observed in ΔD_m pooled across virus triplets with variable backgrounds.

We also performed the triplet analyses on other sites in E and in NS2A. We found significant effects (p<=0.05) for 1 of 8 substitutions in EDI/II/III but outside of known mAb epitopes (S169P, S14 Fig), 0 of 1 substitutions in stem/anchor of E (S15 Fig) and 2 of 20 substitutions in NS2A (L19F and C41L, S16 Fig). The NS2A substitution C41L is in one of the coevolution hotspots with E, and is within pTMS-2, the region most associated with antigenic effect in our larger model."

Discussion:

"To further evaluate how the observed effects hold across genetic backgrounds, we tested whether viruses with and without identified antigenic determinants in E and NS2A differ in antigenicity in the absence of other sources of antigenically relevant changes, thus drawing on the redundancy in our existing dataset to identify the antigenic effects attributable to single amino acid changes. These analyses are a prerequisite for experimental studies to test individual mutations in a clonal background. Our analysis shows that the background virus is important, suggesting experimental studies to identify substitutions driving antigenic changes should be conducted using infectious clones specific to the virus population under study. As designing infectious clones for flaviviruses is difficult, these substitutions provide best candidates for extensive studies to uncover molecular mechanisms underlying the relationship between these substitutions and changes in antigenicity of DENV."

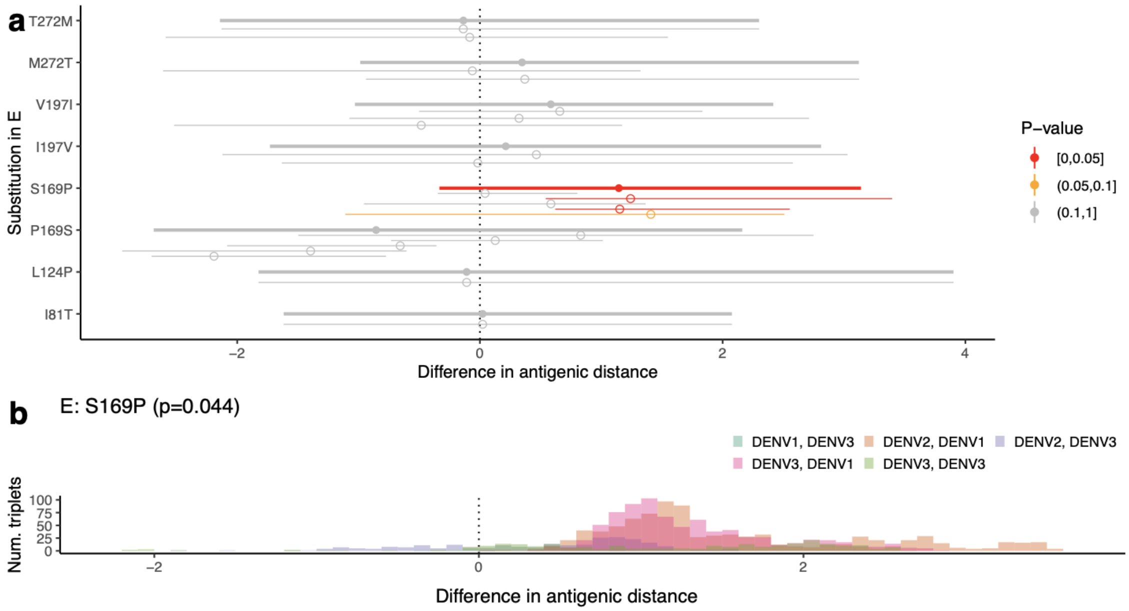

S14 Fig. Effects of substitutions in EDI/II/III but outside of known mAb epitopes. **a)** Difference in antigenic distance observed between pairs of viruses separated by the specific substitution and antigenic distance observed in respective effectively identical viruses without the substitution (control viruses). Thick lines show median and 95% interquartile range (IQR) for triplets of all serotype pairs combined. Thin lines show the median and 95%IQR for each serotype pair identified. **b)** Distribution of difference in antigenic distance for substitution with p-value ≤ 0.1 colored by serotypes of the virus pairs.

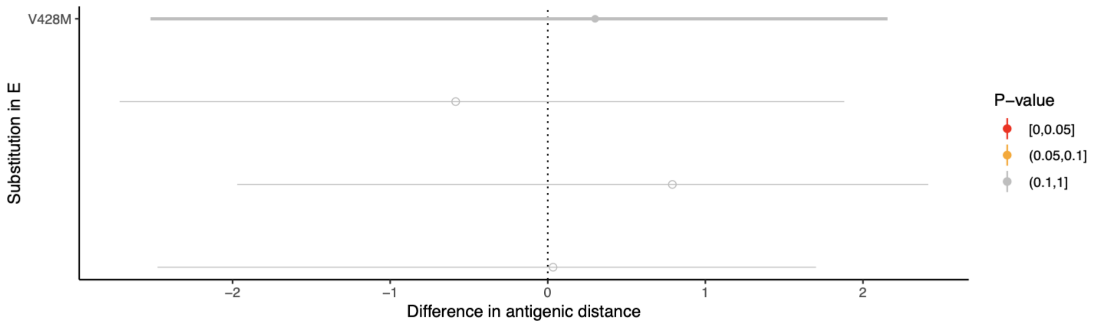

S15 Fig. Effects of substitutions in the stem/anchor domain of E. Difference in antigenic distance observed between pairs of viruses separated by the specific substitution and antigenic distance observed in respective effectively identical viruses without the substitution (control viruses). Thick lines show median and 95% interquartile range (IQR) for triplets of all serotype pairs combined. Thin lines show the median and 95%IQR for each serotype pair identified.

2. It's unclear how amino acid residues in NS2 would modulate antigenicity of dengue viruses. While the authors show statistically significant data in their nonzero sum size model, and speculate in the discussion of likely mechanisms underlying these mutations and their interactions with capsid and prM, these amino acid residues need to be validated through the generation of mutant NS2 viruses to demonstrate if the reversion of the major NS2 hits have a differential phenotype in terms of 1) antibody immune evasion, 2) viral infectivity, or 3) global conformational changes in antigenicity.

Response: We share the view of the reviewer that it is important to evaluate the individual contribution of NS2A residues on our empirical measures of antigenic effects. We have added an additional analysis that takes advantage of the existing diversity present in our dataset. The method is as detailed in the response to your first comment on validating effects of residue changes outside of known mAb epitopes. Results specific to NS2A are as follows.

> *"We also performed the triplet analyses on other sites in E and in NS2A. We found significant effects ($p \leq 0.05$) for … 2 of 20 substitutions in NS2A (L19F and C41L, S16 Fig). The NS2A substitution C41L is in one of the coevolution hotspots with E, and is within pTMS-2, the region most associated with antigenic effect in our larger model."*

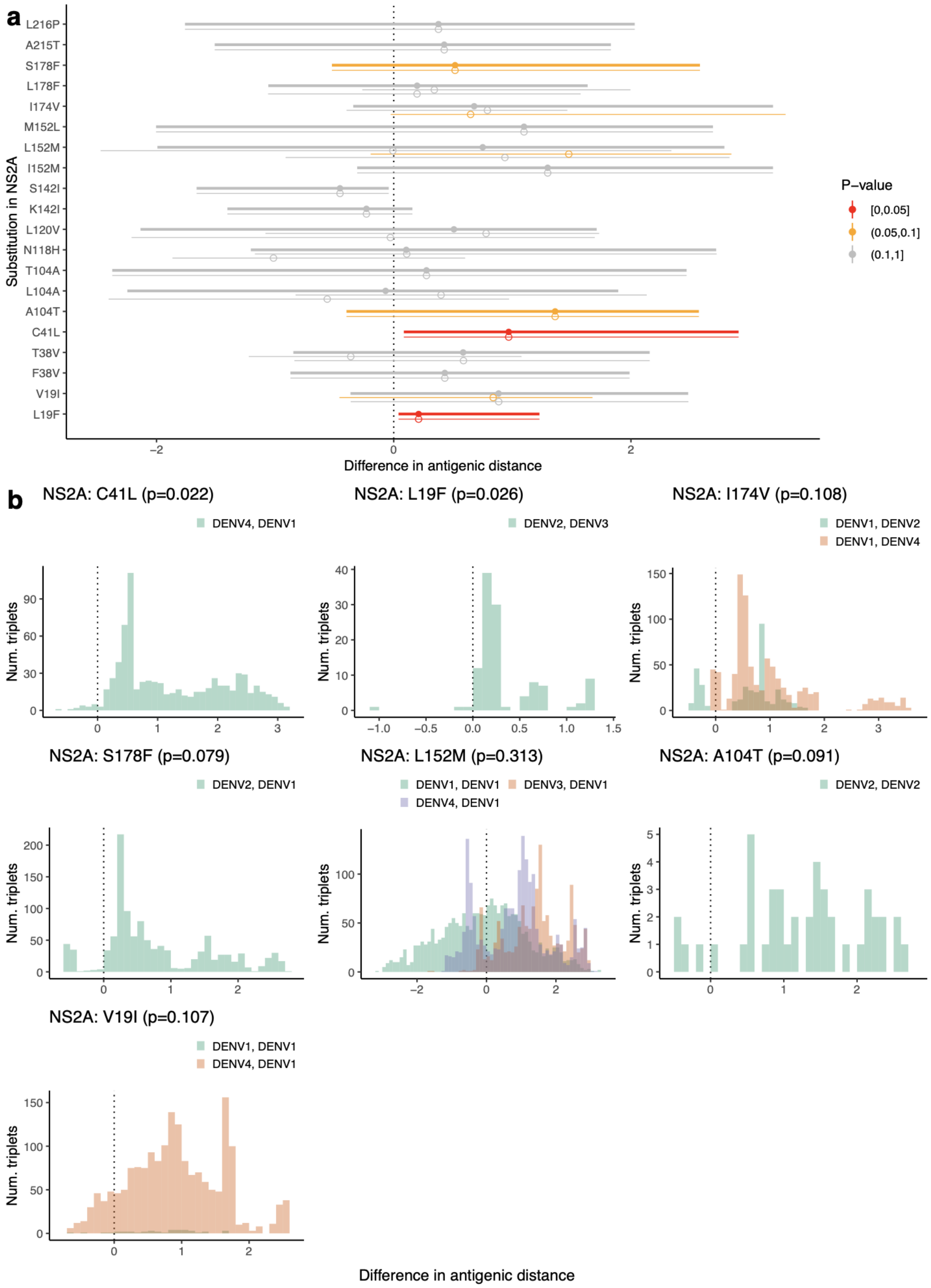

S16 Fig. Effects of substitutions in nonstructural protein 2A (NS2A). a) Difference in antigenic distance observed between pairs of viruses separated by the specific substitution and antigenic distance observed in respective effectively identical viruses without the substitution (control viruses). Thick lines show median and 95% interquartile range (IQR) for triplets of all serotype pairs combined. Thin lines show the median and 95%IQR for each serotype pair identified. **b)** Distribution of difference in antigenic distance for substitutions with p-value ≤ 0.1 colored by serotypes of the virus pairs.

3. Are the authors correcting for multiple comparisons in their statistical analyses? It's not clear from their methods if this is being done. As some of their p values are borderline significant, I suspect they will not be significant after correcting for multiple comparisons as they should do for rigor.

Response: Thank you for the comment. We have added a section to the methods describing our results with increased significance criteria:

> ***Assessing sensitivity of effect determination threshold***
> *Corrections for multiple comparisons involve adjusting the stringency of significance thresholds [52]. We counted the number of estimations that each substitution showed nonzero effect and divided the count by the number of estimations at which effect size estimation of the substitution was attempted to obtain the proportion of estimations in which substitutions showed nonzero effect. We examined the change in number of substitutions with significant effects as we increased the threshold proportion.*

Results from the assessment were included as follows.

> *The number of substitutions identified and number of sites involved changed minimally when we only considered effects present in 100% of estimations as antigenically relevant (S5 Fig).*

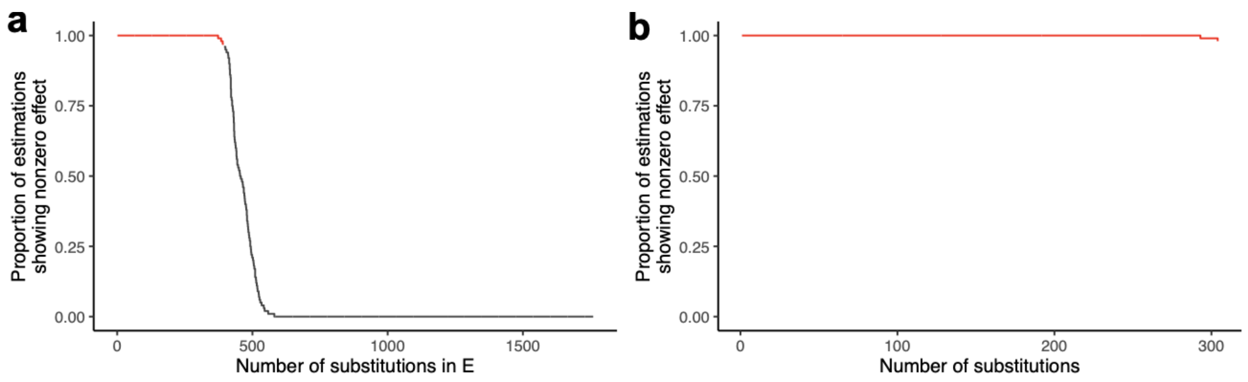

S5 Fig. Proportion of estimations in which substitutions showed nonzero effect. a) Substitutions in envelope protein (E) only, ordered by the proportion at which substitutions showed nonzero effect across the 100 estimations. Substitutions identified by our threshold of 95% was highly similar to the maximum stringency of 100%; 372/394 substitutions (94.4%). Involvement was retained in 76/77 (99%) of the sites. **b)** In the analysis where E was concatenated to the 62 nonstructural protein 2A (NS2A) sites which consistently showed nonzero effects in our site sampling analysis, 292/304 substitutions (96.1%) in the NS2A sites remained nonzero at a threshold of 100%. Involvement was retained in 62/62 (100%) of the sites. Proportions corresponding to nonzero effect substitutions reported in our study (threshold of 95%) are colored red.

Part III – Minor Issues: Editorial and Data Presentation Modifications

Please use this section for editorial suggestions as well as relatively minor modifications of existing data that would enhance clarity.

Reviewer #1: 1. I applaud the authors for the brevity of their manuscript. However, the methods section was quite short, and at times difficult to decipher exactly what the authors did. I would suggest adding the following pieces of information into the methods to help readers who are not familiar with Bell et al.

- The authors should define what the hyperparameters are and why they are set to those values.

Response: Thank you for the suggestions. We have expanded our methods section to explain each of our analyses in detail. In response to your specific points, we replaced “hyperparameters” with “parameters”, a term that is more familiar to the general audience, to avoid unnecessary confusions. In addition to citing the source of the values, we further provided intuition on what those values mean. The revised text is as follows:

> *Weights of these regularization terms were governed by three parameters which were set to the values used in Bell et al., $\lambda = 3.0, \kappa = 0.6, \delta = 1.2$, where the relatively high value of disfavors attributing effects to substitutions, reducing the chance of false attributing effects to substitutions. Results were shown to be insensitive to these values [23].*

- The authors should make clear why 10% of measurements are being withheld. I assumed that this was because 90% of the measurements were used as training data, leaving the remaining 10% as test data, but a sentence explicitly clarifying this would be helpful.

We revised our text on the amount of measurements used in training and testing to the following to make explicit the use of each portion.

> *Effect size estimations were repeated 100 times, including random 90% of the virus pairs each time. The 10% held out were used to test the performance of each estimation. …Root mean squared error (RMSE) evaluated using the test sets were used to describe the prediction performance of the fits.*

- I had to read the Methods section of Bell et al to fully understand their model, and I would guess that other readers of Plos Pathogens would need to do the same. In Bell et al, Dij is connected to dm, vi, and pj, which represent virus avidity, serum potentcy, and the titer drop between viruses. Seeing the explicit connection of Dij to these values made it easier to understand how the effects of each individual mutation was estimated in the model, and the

authors should add it. Currently, it is difficult to figure out how each individual effect is being estimated, given that the only parameter present is Dij, which represents (as I understand it) the sum of all mutations' effects. I suggest the authors add more explicit definitions in their model, including the connection of Dij to dm, vi, and pj.

Response: Thank you for the suggestion. In the Methods, we revised the model explanation paragraph to make explicit the connection of D_{ij} with d_m, v_i, and v_j.

> ***Substitution effect size estimation***
> *We adapted the substitution model described in Bell et al. [23] to analyze the data in our study.*
>
> $$D_{ij} \approx \hat{D_{ij}} = \sum_m d_m + v_i + v_j$$
>
> *Our model approximates the observed antigenic distance D_{ij} between virus i and virus j to the predicted antigenic distance $\hat{D}_{ij}$. The predicted distance is a sum of effects of all substitutions present between the two viruses, $\sum_m d_m$ where d_m is the effect of a single substitution m, and virus-specific measurement uncertainties, v_i and v_j. For a pair of identical viruses, $\sum_m d_m = 0$, any antigenic distance observed between them equals to $v_i + v_j$.*

2. I was a tad confused in the manuscript about how exactly the predictions they perform were being done. From my understanding, the authors built these antigenic maps, then estimated the effects of individual amino acid changes on those distances. However, the authors then describe predicting antigenic distances. Does this mean that the authors estimated antigenic distances with antigenic cartography, then estimated the effects of each individual amino acid change using the modified Bell et al model, then used that information to predict the combined antigenic effect of all amino acids for the strains that did not have PRNT data? Did the authors do this separately for each individual protein sequentially? A paragraph in the methods about how exactly these predictions were done, on which strains, and using data from which genes/ORFs would be helpful.

Response: Thank you for pointing out places where the explanation of our analyses requires further clarification. Yes, that is exactly right. We added a Performance of antigenic distance predictions section to the Methods to provide both descriptions to how our predictions were made and how the performance metric was calculated:

> ***Performance of antigenic distance predictions***
> *We evaluated the performance of the model separately for predicting antigenic distances*

based on mutations in the E protein, each DENV protein, and each DENV protein concatenated to E, and within NS2A. For each of the 100 estimations, we predicted the antigenic distances for the 10% of virus pairs held out during the estimation process. To estimate predicted antigenic distances, where the virus specific intercepts are not known, we sum the effects of the substitutions separating them and adding twice the mean per-virus intercept to the sum. We compute the root mean squared error (RMSE) between predicted distances and antigenic distances derived from the 3-dimensional antigenic map. We report the median and 95% interquartile ranges (IQR) across the 100 estimations.

We added the following text to make explicit which protein sequences were used in each section.

Lines 77-78: *"Using E protein sequences as input, distribution of estimated virus-specific intercepts were similar across the 100 estimations…"*

Lines 145-146: *"To identify antigenic determinants in proteins other than E, (1) we fitted effect sizes for each of the DENV proteins separately, (2) we screened for proteins with predictive performance exceeding that of sites in E,..."*

3. For the last paragraph in the first section, there isn't any data shown. It would be good to add the actual data as a plot showing the correlation between models fitted to E and observed distances.

Response: Thank you for the suggestion. We added S3 Fig to show the association between the fitted and the observed distances:

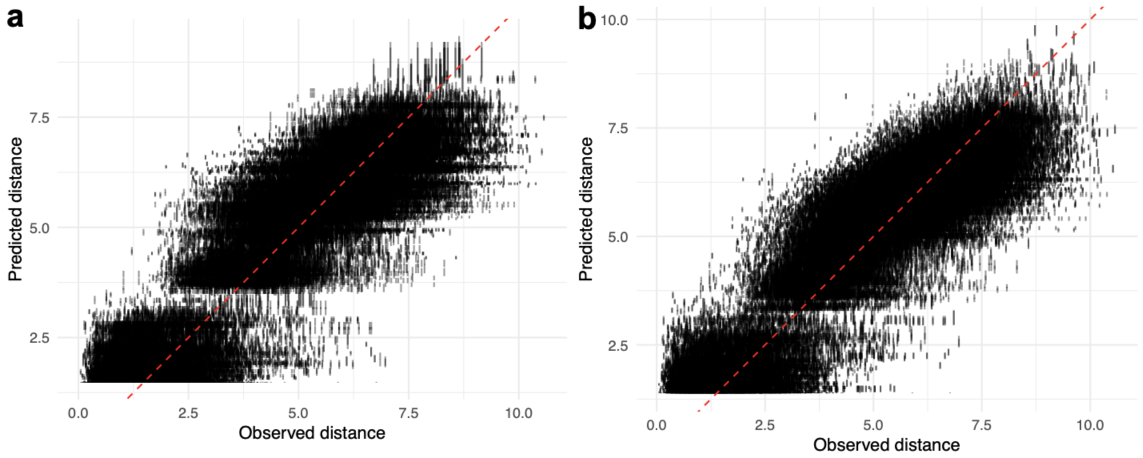

S3 Fig. Relationship between observed antigenic distance and antigenic distance predicted by the substitution model a) when effects were fitted to envelope protein sequences (E) and **b)** when effects were fitted to E concatenated with 62 nonzero effect sites in nonstructural protein 2A (NS2A).

4. The authors write on line 76, "The model identified 394 nonzero effect substitutions positioned on 77 of the 295 sites...". Later, on line 85, they write "158 positions in the E protein contribute to epitopes of characterized anti-DENV mAbs while 336 positions...". Are the authors referring to amino acid sites in 1 part, and nucleotides in the other? Are they referring to different proteins? I was confused about why the denominator for the number of sites on E is different in these 2 sentences.

Response: Thank you for the comment. In the latter paragraph, we opened with describing the DENVab database as we expect not all readers will be familiar with it. To mitigate the confusion, we added the following text to make explicit that the number of sites reported from the DENVab database may exceed the number of sites detectable in our dataset due to absence of variability.

> *According to DENVab database, 159 positions in the E protein contribute to epitopes of characterized anti-DENV mAbs while 336 positions have not yet been associated with any epitopes. Seventy of the mAbs were recorded to have neutralization activity, footprints involving 111 sites. Of these, 74 sites were variable in our dataset meaning their effects have the potential to be detected by the model.*

5. Figure 3 is quite blurry and a bit difficult to read. Figure 3d especially is difficult to interpret because all of the points are overlapping. Perhaps a histogram would help in showing the bimodal distribution? As is, every site looks the same, and it is impossible to distinguish how many sites have 0 vs. 1 effects.

Response: Thank you for the suggestion. We now separate this large panel plot into three figures (new Fig 4, Fig5 and S6 Fig) to improve visibility of features in the plot. We replaced panel d with histograms as suggested and added lines between them to link frequencies of being estimated as nonzero effect of the same positions.

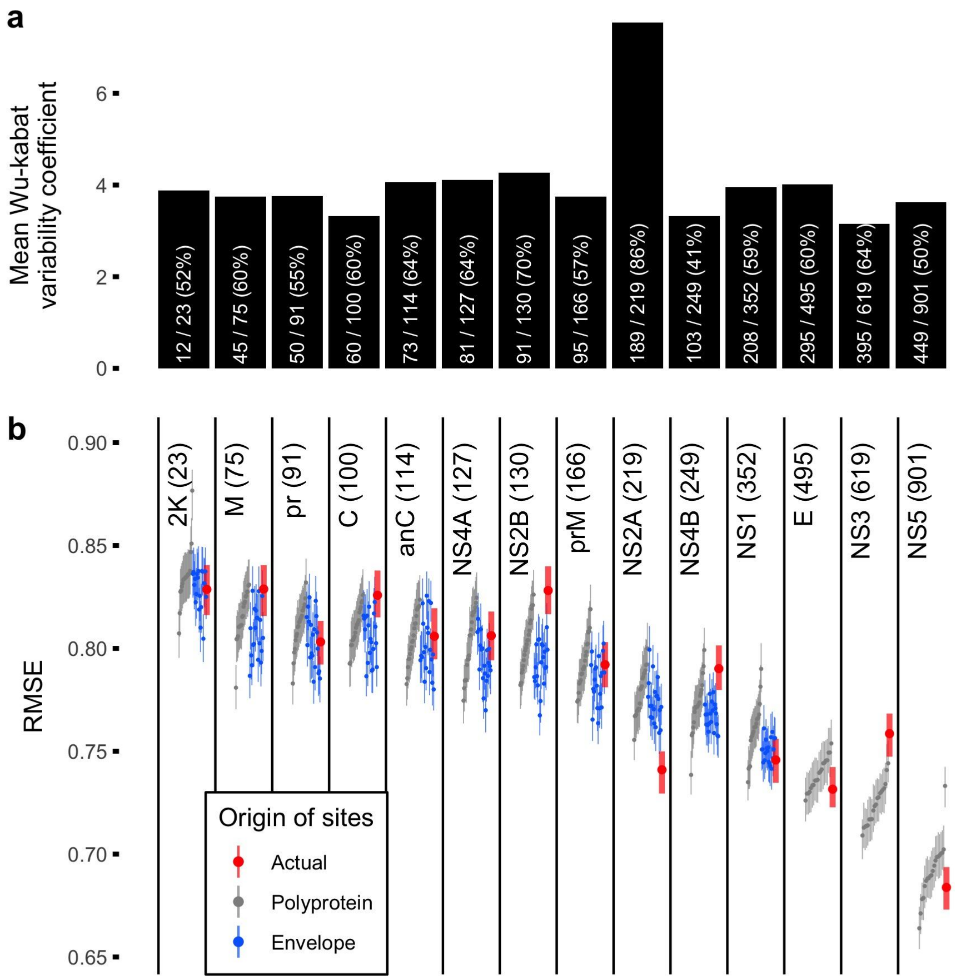

Fig 4. Antigenic signal in each DENV protein. a) Average within site variability in DENV proteins observed in the dataset. Bars were annotated with number of variable sites, total number of sites, and percentage of sites variable. **b)** Prediction performance of each DENV protein as observed (red) contrasted against expectations derived from random subsample of sites from any DENV protein of the same length (gray) and random down samples of sites from the envelope protein (E, blue). Points and lines are median and 95% interquartile range (IQR) of the root mean squared error (RMSE) evaluated under 100-fold Monte Carlo cross-validation. Length of the proteins are shown in parentheses. Only nonstructural protein 2A (NS2A) appeared to have better predictive performance than the expectations.

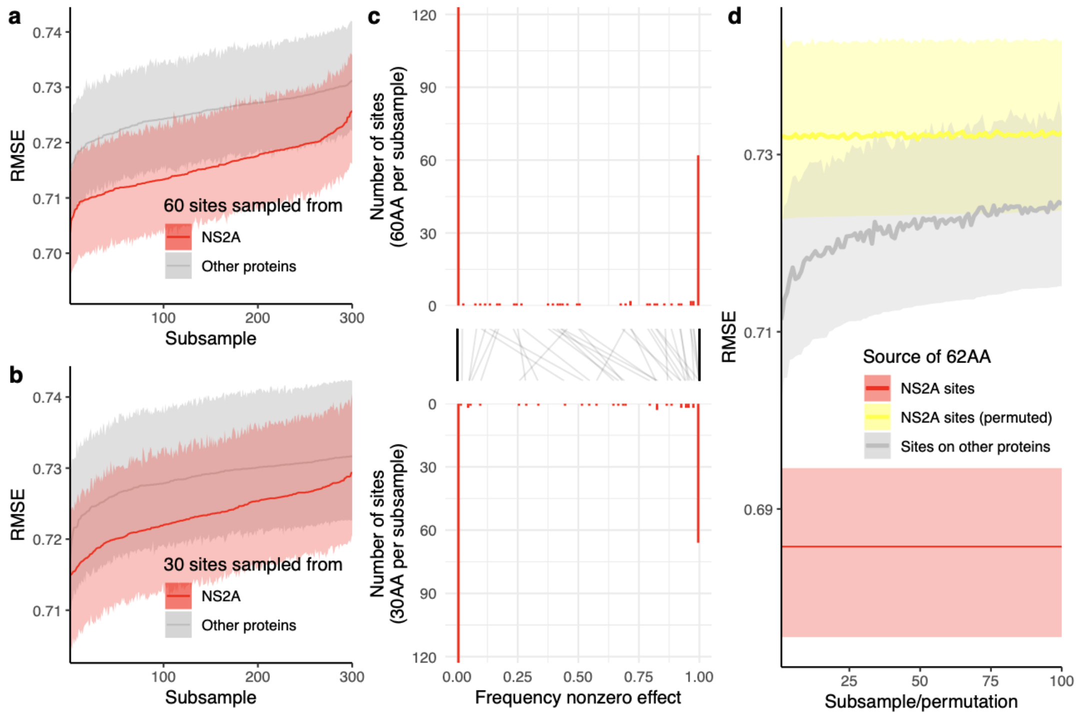

Fig 5. Sites embedding antigenic signals beyond the envelope protein. Prediction performance of downsampled NS2A sites concatenated with E when randomly downsampled to **a)** 60 sites and **b)** 30 sites contrasted against when concatenated with random sites from other proteins. **c)** Distribution of frequencies at which sites showed non-zero effect given being sampled in the two downsampling schemes. Black lines link frequencies of the same sites. **d)** Performance when concatenating the 62 sites which >99% of the times sampled was estimated to have non-zero effect size when adjusted for E in both schemes (red) compared against the same sites but permuted (yellow), and sites from other proteins of the same length (gray). Permutation was done by permuting residues observed at each site across viruses to conserve its diversity.

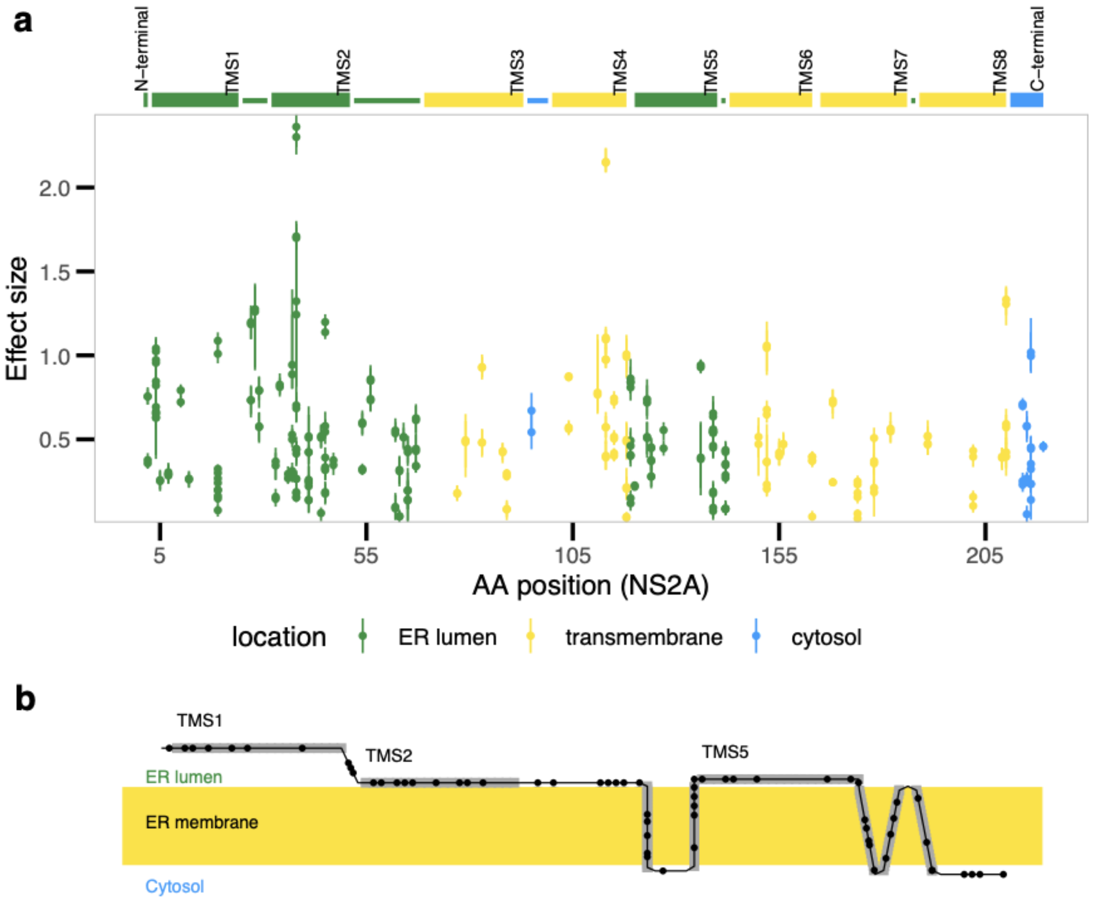

S6 Fig. Substitutions with non-zero effect sizes in NS2A. a) Median effect size of substitutions across the 100-fold Monte Carlo cross-validations shown as points, 95\% interquartile range as whiskers. Points are colored by locations of the sites: ER lumen (green), transmembrane (yellow), or cytosol (blue). Locations of the sites and domain annotations were taken from Xie, 2013. **b)** Topological diagram of NS2A adapted from Xie, 2013 with the 62 sites marked as points. Predicted transmembrane segments (pTMS) shaded in gray.

6. In Figure 2c, how do the authors interpret that their model estimated 0 effects for 1/4 of the known epitopes? Similarly, it seems like their model was equally likely to estimate 0 vs. non-0 effects for known epitopes. Why do they think this is?

Response: Thank you for the comment. In response to another reviewer, we revisited our analyses restricting to only footprints of human-derived mAb. In doing so, the association between model estimated nonzero effect sites and human-derived mAb epitopes improved in both odds ratio and neighborhoodness. These new results and our interpretation of our model estimates have been added to the main text as follows.

Results: *"Interestingly, while 36 sites previously identified as DENV-specific hmAb epitopes were marked as zero-effect size by the model, 29 sites (80.5%) were within 3 linear positions away from a nonzero effect residue. In reverse, of the 56 nonzero effect sites that did not match the reported hmAb epitopes, 28 were within 3 sites of known hmAb epitopes, suggesting that they plausibly could contribute to epitopes for some previously identified antibodies. The chance of observing at least this amount of overlap, 21 captured + 28 proximal, if 77 sites were chosen from the 295 sites with variability at random was small (p=0.037, Fig 2b). We repeated the analysis using distances extracted from a resolved 3-dimensional structure of E [28]. The chance was also small when proximal sites were defined as being within 3.5 angstroms away (p=0.014, Fig 2c)."*

Discussion: *"Our studies of the E protein suggest our model is likely conservative in attributing effects to sites/substitutions and is returning hits more specific to antibody responses in primates. Of the sites on the E protein marked as antigenically relevant by our model, 63.6% were within or neighborhooding known human epitopes but not mouse epitopes. This association was greater than random chance within 3 positions or 3.5 angstroms around known epitopes. Of the remaining antigenically relevant sites, 16/28 were in the stem/anchor domains, which have recently been shown to become exposed under physiological conditions but mAb targeting these sites have yet been identified. These comparisons provide support for antigenic signal in sites as measured by polyclonal responses, which may be similar to identified monoclonal antibodies but may target the same antigenic regions in a slightly different way. Alternatively, some of the sites we identified were not near known epitopes. Our findings suggest that polyclonal antisera may target epitopes beyond those of currently identified monoclonal antibodies and also support recent studies showing that changes at specific sites may introduce global changes to the virus that affect polyclonal neutralization in a non-epitope specific manner."*

7. In sections 110-115, the authors describe that individual gene trees match full genome trees, which would make sense if there is little recombination in dengue viruses. It would be nice to explicitly acknowledge whether dengue viruses recombine, add a reference, and directly acknowledge how their test accounts for that.

Response: Thank you for this suggestion. We added text and references as follows to point to available reports of recombination in dengue viruses. We also added discussion around our anticipated effects of recombination on our results as follows.

Results: *"Phylogenies inferred from individual genes were shown to have branching patterns similar to ones inferred from the complete genome or the open reading frame (ORF), with nonstructural genes, except NS4A, yielding better resolution (i.e., stronger clade support values) than structural genes [30–32] despite some reports of DENV intraserotype recombination [33]."*

Discussion: *"Also, we did not account for recombinations between DENV, which has been reported to occur within serotypes between homologous sites [33]. Although this complicates phylogenetic reconstructions, our model is unlikely to be affected by recombination as it is phylogeny-free. In fact, presence of recombination accelerates the dissolve of linkage between sites, increasing diversity of sequence combinations, which makes effects of individual substitutions more likely to be detected."*

Reviewer #2: 1. Huang et al. found 394 substitutions with nonzero effect on 77 residues from the E protein. Notably, only 22 of them (~28%) mapped to known epitopes. While this information is presented in Fig 2, the exact location is missing. This data is very interesting and could inform other studies, so authors should provide a supplementary table with the list of all those 77 residues and describe which ones map on known epitopes.

Response: Thank you for the suggestion. To ease data extraction for enthusiastic readers, we provided the positions in the accompanying data repository as CSV files instead of supplementary PDF documents. We now reference the supplemental data file in the Results:

Lines 91-93: *"The model identified 394 nonzero effect substitutions positioned on 77 of the 295 sites on the E protein with residue diversity observed in the Thai DENV dataset (Fig 2, S4 Fig, S1 File)."*

Line 191: *"The 62 sites identified in NS2A (S2 File) were scattered throughout the protein …"*

2. The model predicted that over 2/3 (52/74) of the residues that presented variability and mapped to known epitopes were not involved in antigenic differences (zero effect size, Fig 2). Please specify whether those residues are (mostly) associated to residues mapped with non-human mAbs.

Response: Thank you for your suggestion. We tabulated the relationship between zero (or non-zero) effect sizes against sources of mAb and found that when only considering footprints from human mAbs as known epitopes, association between our predicted effects and known epitopes improved (odds ratio of 1.90, 95%CI: 1.02, 3.51). Revisiting our neighboring sites analysis, we found a stronger relationship between nonzero effect sites determined by our model and epitopes when limited to human mAbs (Fig 2). We added these findings to our results and discussion as follows.

Results:

"Interestingly, while 36 sites previously identified as DENV-specific hmAb epitopes were marked as zero-effect size by the model, 29 sites (80.5%) were within 3 linear positions away from a nonzero effect residue. In reverse, of the 56 nonzero effect sites that did not match the reported hmAb epitopes, 28 were within 3 sites of known hmAb epitopes,

suggesting that they plausibly could contribute to epitopes for some previously identified antibodies. The chance of observing at least this amount of overlap, 21 captured + 28 proximal, if 77 sites were chosen from the 295 sites with variability at random was small (p=0.037, Fig 2b). We repeated the analysis using distances extracted from a resolved 3-dimensional structure of E [28]. The chance was also small when proximal sites were defined as being within 3.5 angstroms away (p=0.014, Fig 2c)."

Discussion:

"Our studies of the E protein suggest our model is likely conservative in attributing effects to sites/substitutions and is returning hits more specific to antibody responses in primates. Of the sites on the E protein marked as antigenically relevant by our model, 63.6% were within or neighborhooding known human epitopes but not mouse epitopes. This association was greater than random chance within 3 positions or 3.5 angstroms around known epitopes. Of the remaining antigenically relevant sites, 16/28 were in the stem/anchor domains, which have recently been shown to become exposed under physiological conditions but mAb targeting these sites have yet been identified. These comparisons provide support for antigenic signal in sites as measured by polyclonal responses, which may be similar to identified monoclonal antibodies but may target the same antigenic regions in a slightly different way. Alternatively, some of the sites we identified were not near known epitopes. Our findings suggest that polyclonal antisera may target epitopes beyond those of currently identified monoclonal antibodies and also support recent studies showing that changes at specific sites may introduce global changes to the virus that affect polyclonal neutralization in a non-epitope specific manner."

3. Authors should provide a better description on how the 348 viruses isolated (18% from total) were selected for this study. This reviewer needed to go back to the recently published paper from this group (Katzelnick et al. Science 2021) to gather more information on the distribution of serotypes and genotypes for this study. This could be included as additional panel for Fig 1.

Response: Thank you for the suggestion. We now include the genotypes of the viruses in the Data section of Material and methods and have added a phylogenetic tree showing the viruses selected for antigenic characterization.

"Our study utilized whole genome sequences and 3-dimensional antigenic map coordinates of 348 DENV previously characterized by Katzelnick et al [22]. In brief, 1,944 isolated viruses were derived from serum specimens collected from acute illnesses admitted to the Queen Sirikit National Institute of Child Health (QSNICH) in Bangkok, Thailand, mostly between 1994 and 2014. Aside from a genotype replacement of DENV3 from genotype II to genotype III, viruses were primarily of a single dominant genotype for each serotype (DENV1 genotype I, DENV2 genotype Asian I, DENV4 genotype I). From the 1,944 whole genome sequences acquired (667 DENV1, 440 DENV2, 454 DENV3, and 383 DENV4), the isolates were systematically sampled to

represent amino acid variations in the envelope (E) protein and pre-membrane (prM) protein and to balance across all years between 1994 and 2014, resulting in 348 virus isolates (18%; 87 DENV1, 80 DENV2, 90 DENV3, and 91 DENV4) being antigenically characterized."

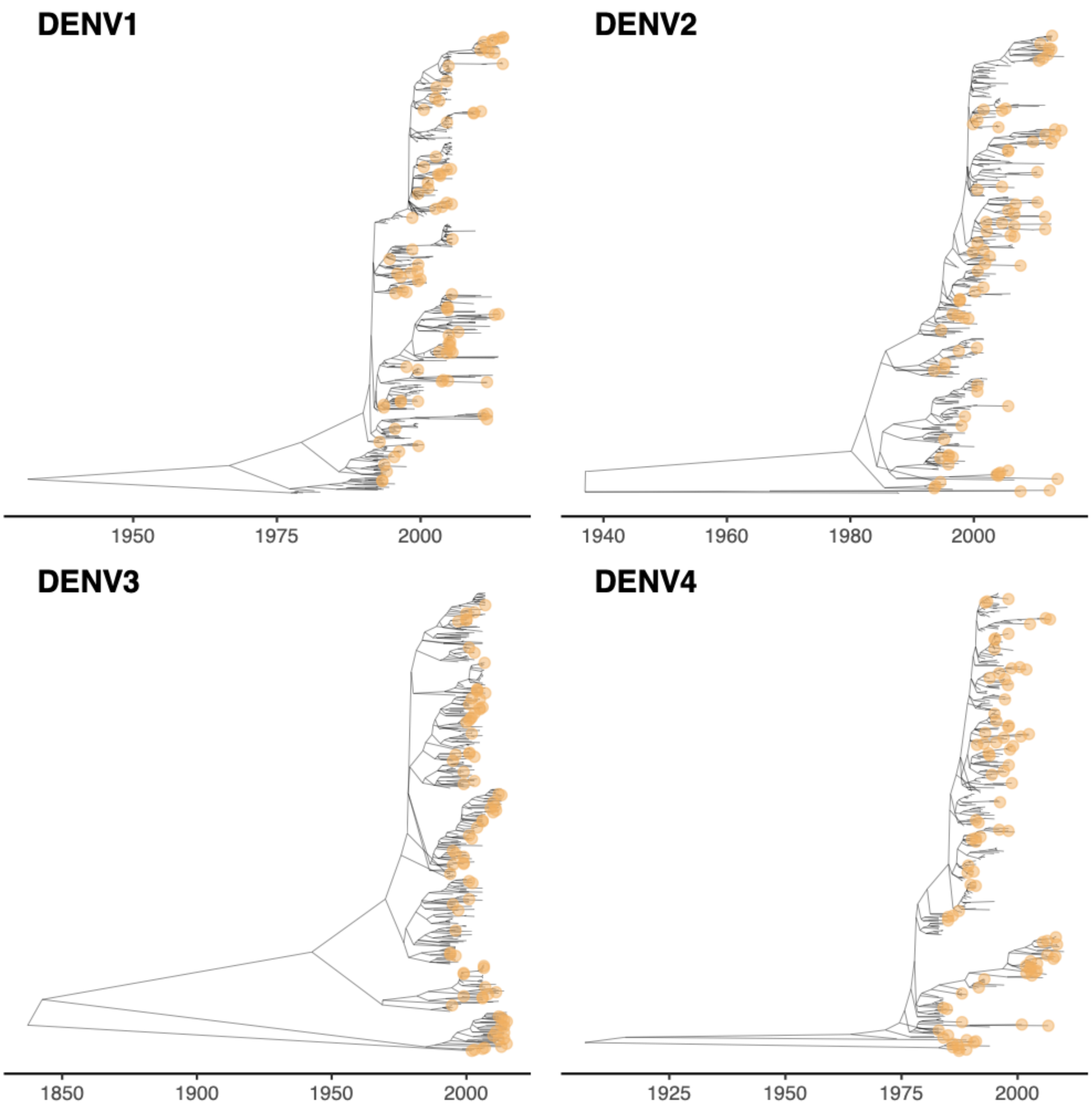

S1 Fig. Time-calibrated maximum likelihood phylogenies of virus isolates collected from Queen Sirikit National Institute of Child Health (QSNICH) between 1994-2014. Viruses selected for antigenic characterization were marked as orange circles.

4. Figure S2 should be plotted to summarize the data. It is hard to determine what proteins of dengue are the most variable. Moreover, authors stated that "295 site on the E protein" presented residue diversity in the Thai dataset, however, it is difficult to determine that number

from the current figure. Please considering including this (revised) data as part of the main manuscript.

Response: Thank you for the suggestion. We added the suggested plot to Fig 4 and included annotations to provide the number of sites (and percentages) variable in each protein.

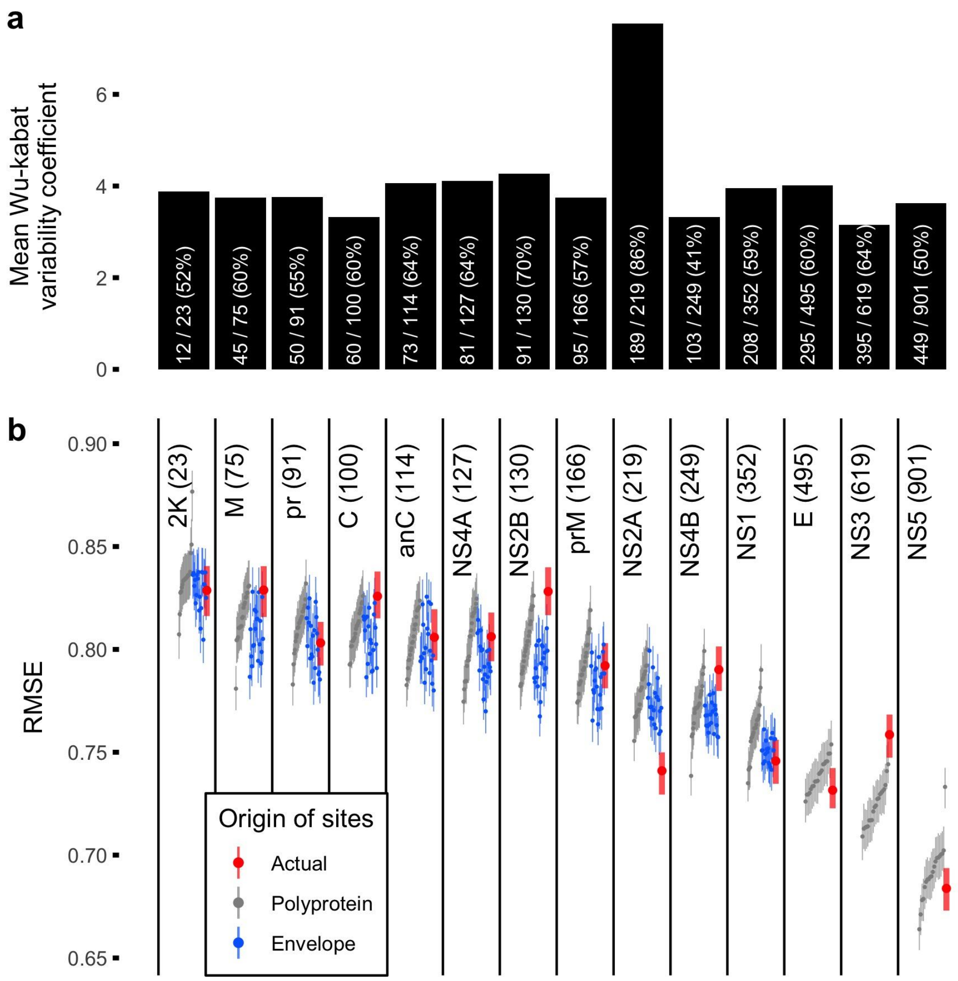

Fig 4. Antigenic signal in each DENV protein. a) Average within site variability in DENV proteins observed in the dataset. Bars were annotated with number of variable sites, total number of sites, and percentage of sites variable. **b)** Prediction performance of each DENV protein as observed (red) contrasted against expectations derived from random subsample of sites from any DENV protein of the same length (gray) and random down samples of sites from the envelope protein (E, blue). Points and lines are median and 95% interquartile range (IQR) of the root mean squared error (RMSE) evaluated under 100-fold Monte Carlo cross-validation. Length of the proteins are shown in parentheses. Only nonstructural protein 2A (NS2A) appeared to have better predictive performance than the expectations.

5. Lines 96-97: I believe the authors meant “41 residues (78.8%)”

Response: Thank you for bringing to our attention the inclarity in this part of our text. We rephrased the statement to the following for better clarity. Of note, the numbers have changed as we now focus specifically on overlap with hmAb epitopes.

> *"Interestingly, while 36 sites previously identified as DENV-specific hmAb epitopes were marked as zero-effect size by the model, 29 sites (80.5%) were within 3 linear positions away from a nonzero effect residue."*

6. Lines 172-173: Please provide the reference.

Response: Thank you for pointing this out. We have added the reference to make clear that both sentences were results from Nemeśio and Villalaín.

> *"With its characterized properties, Nemeśio and Villalaín [35] speculated that it has a role in membrane rearrangements during replication."*

Reviewer #3: 1. The first sentence in the first abstract is inaccurate: "Neutralizing antibodies are important correlates of protection against dengue virus (DENV) infections." What is known is that neutralizing antibodies are associated with protection from severe DENV disease. However, it is not known if neutralizing antibodies can prevent subclinical viral infections that are asymptomatic. The authors should change sentence for factual accuracy or provide conclusive data that states otherwise.

Response: We agree that neutralizing antibodies are correlated with protection against dengue. We revised the abstract to read:

> *"Neutralizing antibodies are important correlates of protection against dengue. Yet, determinants of variation in neutralization across strains within the four dengue virus serotypes (DENV1-4) is imperfectly understood."*
